# Associations of Fructose Consumption with Prevalence and Incidence of Metabolic Dysfunction–Associated Steatotic Liver Disease—The Kuopio Ischaemic Heart Disease Risk Factor Study

**DOI:** 10.1016/j.tjnut.2025.101318

**Published:** 2026-01-06

**Authors:** Ronja H Saarinen, Heli EK Virtanen, Sari Hantunen, Jukka T Salonen, Tomi-Pekka Tuomainen, Jyrki K Virtanen

**Affiliations:** 1 Institute of Clinical Medicine, University of Eastern Finland, Kuopio, Finland; 2 Institute of Public Health and Clinical Nutrition, University of Eastern Finland, Kuopio, Finland; 3 Department of Public Health, the Faculty of Medicine, University of Helsinki, Helsinki, Finland; 4 MAS-Metabolic Analytical Services Oy, Helsinki, Finland

**Keywords:** fructose, hepatic steatosis, metabolic dysfunction**–**associated steatotic liver disease, population study, sweeteners

## Abstract

**Background:**

Metabolic dysfunction**–**associated steatotic liver disease (MASLD) is the leading cause of liver diseases. Fructose intake has been associated with liver fat accumulation, but less is known whether the associations differ based on the source of fructose.

**Objectives:**

We investigated the cross-sectional and longitudinal associations of intake of total fructose and fructose from different sources with risk of MASLD among middle-aged and older people from Eastern Finland.

**Methods:**

The cross-sectional analyses included 666 males and 865 females aged 53–73 y, examined in 1998–2001. The longitudinal analyses included 300 males and 467 females examined again in 2005–2008. Fructose intake was assessed with 4-d food record. Fatty liver index (FLI) was used as a surrogate for liver fat content. MASLD was defined as FLI ≥60 and the presence of ≥1 cardiometabolic risk factors. Analysis of variance and multivariable-adjusted logistic regression were used for analyses.

**Results:**

The mean total fructose intake was 33 g/d (standard deviation 13.4, 7.4% of the total energy intake), with sweeteners (mainly sugar, 34.5% of the total fructose intake), fruits and berries (20.2%), and beverages (18.9%) being the major sources. In the cross-sectional analyses, participants with higher total fructose intake had 43% lower odds for MASLD [95% confidence interval (CI): 10%, 64%] in those in the highest (>39.7 g/d) compared with the lowest (<24.6 g/d) intake quartile (*P*-trend across quartiles = 0.02). Among the sources of fructose, the strongest inverse associations were observed with fructose from sweeteners. In the longitudinal analyses, total fructose intake was not associated with MASLD. However, fructose from sweeteners again had a strong inverse association with odds for MASLD (78% lower odds in the highest compared with the lowest quartile, 95% CI: 49%, 90%; *P*-trend < 0.001). Fructose from fruits and berries or from beverages was not associated with MASLD.

**Conclusions:**

In middle-aged and older Finnish adults, higher fructose intake, especially from sweeteners, was associated with lower odds for MASLD.

## Introduction

Metabolic dysfunction**–**associated steatotic liver disease (MASLD), formerly known as nonalcoholic fatty liver disease (NAFLD) [1,2], is the leading cause of chronic liver disease worldwide [3,4] and it is estimated that about one-third of the world population has MASLD [4,5]. In contrast to the NAFLD diagnosis, which only required hepatic steatosis, MASLD diagnosis requires liver steatosis together with ≥1 of 5 cardiometabolic risk factors, for example, presence of impaired glucose regulation, type 2 diabetes, overweight or obesity, hypertension, or dyslipidemia [1]. MASLD is associated with hepatocellular carcinoma, cardiovascular disease (CVD), systemic malignancies, and chronic kidney disease [6,7]. MASLD can progress to metabolic dysfunction**–**associated steatohepatitis or even to hepatic cirrhosis. Lifestyle is a significant factor in the progression of metabolic diseases, including MASLD. The current treatment for MASLD is based on lifestyle modification aiming for weight loss and include dietary changes [8].

One nutritional factor that has drawn attention in the development of MASLD is fructose. Abundant fructose ingestion can increase the amount of newly synthesized fatty acids, which are recognized as major contributors to hepatic fat accumulation associated with obesity [9]. Fructose can be found, for example, from sugar-sweetened beverages, candies, honey, and fruits. Sucrose, or table sugar, consists of 1 glucose and 1 fructose molecule. Besides sugar, glucose-fructose-syrup or fructose-glucose-syrup can also be used as sweeteners.

Consuming fructose-sweetened, but not glucose-sweetened, beverages has been shown to increase visceral adiposity, promote dyslipidemia and decrease insulin sensitivity [10]. On the other hand, fructose and glucose in combination might be more harmful than fructose alone [11]. One study showed that consumption of both sucrose and high-fructose corn syrup induced detrimental changes in hepatic lipids, insulin sensitivity, and circulating lipids, lipoproteins, and uric acid [12]. Excess amount of fructose-containing sugars was also found to increase intrahepatocellular lipid and circulating alanine aminotransferase concentrations in hypercaloric trials [13,14]. In 1 study, fructose restriction led to a small decrease in intrahepatic lipid content [15].

However, the results from experimental studies may not be directly generalizable to populations with typical diets, because the amount of fructose in those studies has been much higher than commonly consumed [[12], [13], [14]]. The observational evidence of the associations of fructose consumption with MASLD is limited. The study findings are also mixed, as some studies in adults have found no association between high total fructose consumption and increased intrahepatic lipid content, fatty liver or NAFLD [[16], [17], [18]], but 1 Finnish study found an inverse association with high fructose consumption and prevalence of NAFLD [19]. In pediatric patients, however, a positive association between high fructose intake and NAFLD has been found [20], and 1 study found that lower fructose intake was associated with decreased risk of NAFLD [21]. Even though different food sources of fructose have been shown to have different associations with NAFLD [13,22], there is only little knowledge of how fructose from different sources relates to MASLD/NAFLD. Some studies found that total fructose intake and fructose from fruits were not associated with increased intrahepatic lipid content, but fructose from fruit juices and sugar-sweetened beverages was associated with increased prevalence of intrahepatic lipid content or fatty liver index (FLI) [16,17].

Our purpose was to add new evidence to the limited observational data currently available regarding the role of fructose consumption in the development of MASLD in adults using data from middle-aged and older male and female participants from Eastern Finland. In addition to the cross-sectional analyses, we also investigated the associations of total fructose consumption and fructose from different dietary sources with the development of MASLD during the mean 8 y of follow-up. We hypothesized that higher total fructose intake would be associated with lower odds for MASLD, similar to the other Finnish study [19], but the associations would differ based on the source of fructose. Especially fructose from sweeteners and beverages would be associated with a higher risk.

## Methods

### Study population

The Kuopio Ischaemic Heart Disease Risk Factor Study (KIHD) is a prospective population-based cohort study from Eastern Finland. It was primarily designed to investigate risk factors for CVDs, atherosclerosis, and related outcomes in a population-based sample of males and females from Eastern Finland [23]. Other outcomes, such as MASLD, can be considered as secondary outcomes. The KIHD study adhered to the Declaration of Helsinki, and it has an approval of the Research Ethics Committee of the University of Kuopio. All the participants gave a written informed consent for participation.

**FIGURE 1:**
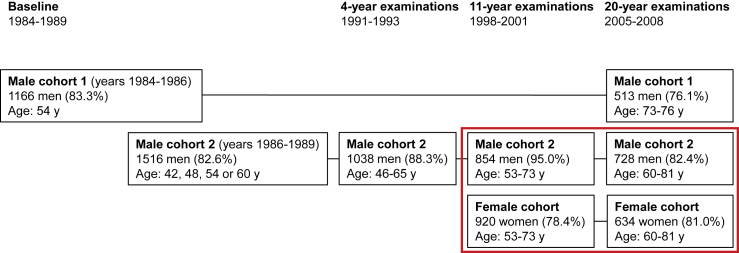
The timeline of the Kuopio Ischaemic Heart Disease Risk Factor Study. The figure shows the total numbers of subjects who participated in each examination round. The percentages in brackets show the proportion of those participants who participated in the examination rounds, among all eligible participants. The red box shows the participants who were included in the current analyses.

The baseline examinations of KIHD were conducted in 1984–1989 on 2 male cohorts, a total of 2682 males aged 42, 48, 54, or 60 y from the city of Kuopio and the surrounding rural neighborhoods (Figure 1). Most of the males from the second cohort were examined again in the follow-up visits in 1991–1993 and in 1998–2001. The examinations in 1998–2001 were also the baseline for a female cohort of 920 postmenopausal females aged 53–73 y from the same area. In 2005–2008 all males, from both baseline male cohorts, and all females were invited for the final KIHD study visit.

**FIGURE 2:**
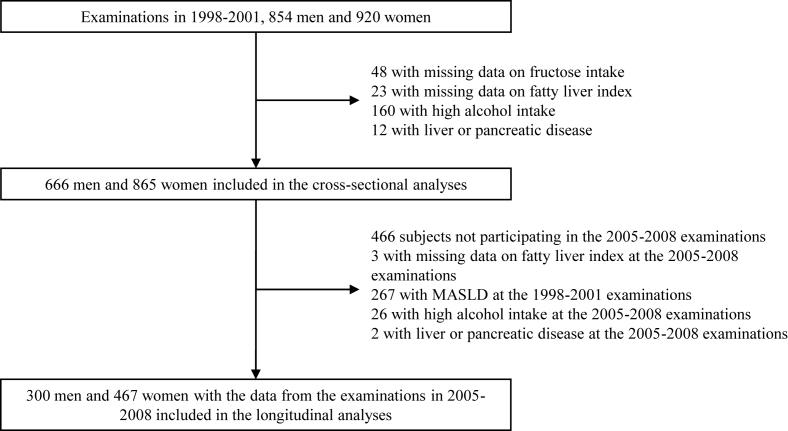
The number of subjects included in the analyses. MASLD, metabolic dysfunction**–**associated steatotic liver disease.

For the analyses of the current study, we used the male and female cohorts examined in 1998–2001 and 2005–2008. We excluded from the analysis all participants with high alcohol intake (>20 g/d) or participants with missing data on nutritional factors or on FLI or who had a diagnosis of liver or pancreatic disease at the examinations (Figure 2). The cross-sectional analyses included 666 males and 865 females, and the longitudinal analyses included 300 males and 467 females.

### Assessment of dietary intakes

Dietary intakes were assessed in 1998–2001 with a food record of 4 d, one of which was a weekend day [24]. A nutritionist instructed participants to record all consumed foods and beverages in household measurement units. A picture book of common foods and dishes in Finland was used to help estimate the portion sizes. A nutritionist checked the completed records together with the participant to further improve the accuracy.

Food and nutrient intakes were calculated from the food records using the NUTRICA 2.5 software (Social Insurance Institution of Finland). The databank of the software is mainly based on Finnish values of nutrient composition of foods. The amount of total fructose was calculated by assessing the amount of sucrose consumed and dividing it by 2 and adding that to the amount of free fructose. Similarly, the total amount of fructose in specific dietary source accounted for the free fructose in the source and a half of the amount of sucrose in the source. All nutrients were energy-adjusted by the residual method [25]. As an indicator of the overall diet quality, the modified Baltic Sea Diet Score was used [26]. The score ranges from 0 to 25, with higher score indicating better adherence to a healthy Nordic diet. In the current analyses, when used as a covariate, the score did not include alcohol intake and thus ranged from 0 to 24. Alcohol component was excluded from the score to prevent overadjustment, as alcohol intake was adjusted for separately.

### Measurements

Venous blood samples were collected between 08:00 and 10:00 at the examinations. Participants were instructed to abstain from ingesting alcohol for 3 d and from smoking and eating for 12 h prior to giving the sample. Detailed descriptions of the determinations of medical history and medications, serum lipids and lipoproteins, serum fatty acids, smoking, alcohol intake, and blood pressure have been published [27,28]. For the current study, medications with potential effects on liver fat accumulation were considered as potential confounders. These included the following drugs that were consumed by ≥1 participant: aspirin, ibuprofen, acetaminophen, corticosteroids, losartan, naproxen, methotrexate, omeprazole, pentoxifylline, valproic acid, amiodarone, tamoxifen, and tetracycline [29]. Serum gamma-glutamyl transferase (GGT) activity was measured using the kinetic method (Thermo Fisher Scientific). The lipoprotein fractions were separated from serum using ultracentrifugation and precipitation. The triglyceride contents of lipoprotein fractions were measured enzymatically (CHOD-PAP method, Boehring Mannheim). Hypertension was defined as blood pressure >140/90 mmHg or medication for hypertension. Diabetes was defined as a self-reported physician-set diagnosis of diabetes and/or fasting plasma glucose ≥7.0 mmol/L or 2-h oral glucose tolerance test plasma glucose ≥11.1 mmol/L. The metabolic syndrome was defined as the presence of 3 or more of the following: *1*) fasting plasma glucose ≥5.6 mmol/L; *2*) serum triglycerides ≥1.7 mmol/L; *3*) serum HDL <1.0 mmol/L; *4*) blood pressure ≥130/85 mmHg or use of blood pressure medication; or *5*) waist circumference >102 cm (males) or >88 cm (females).

Waist circumference, weight, and height were measured at the study visit by a study nurse. BMI was calculated as the ratio of weight in kilograms to height in meters squared (kg/m^2^). Physical activity was evaluated based on the 12-mo leisure-time physical activity questionnaire. The questionnaire was used to record the average duration, intensity, and frequency of the most common leisure-time physical activity types, and this information was used to calculate energy consumption in kcal/d [30]. Education was assessed in years by using self-administered questionnaire.

### Diagnosis of MASLD

**FIGURE 3:**
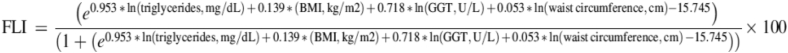
Mathematical formula for the FLI. FLI, fatty liver index; GGT, gamma-glutamyl transferase.

For defining hepatic steatosis, we used FLI, a mathematical formula that predicts the presence of liver fat. FLI is based on BMI, waist circumference, and serum triglyceride and GGT concentrations (Figure 3). FLI <30 rules out and FLI ≥60 rules in hepatic steatosis [31].

MASLD diagnosis was defined as FLI ≥60 and presence of at least one of the following cardiometabolic risk factors: BMI ≥ 25 or waist circumference >94 cm (males) or 80 cm (females), fasting plasma glucose ≥5.6 mmol/L or 2-h oral glucose tolerance test glucose ≥7.8 mmol/L or diagnosis of type 2 diabetes or treatment for type 2 diabetes, blood pressure ≥130/85 mmHg or use of antihypertensive medication, serum triglycerides ≥1.70 mmol/L or serum HDL cholesterol ≤1.0 mmol/L or use of lipid lowering medication [1].

### Statistical analysis

Data were analyzed with SPSS (Statistical Package for the Social Sciences) version 29 software (IBM Corp.). The univariate associations of fructose intake with FLI were estimated with analysis of variance. Logistic regression in quartiles of fructose consumption and per 5 g increase in intake was used to estimate the odds for prevalence and incidence of MASLD. Linear trends across fructose intake quartiles were assessed after assigning the median value for each intake quartile and then treating that as a continuous variable in linear regression.

The confounders were selected based on established risk factors for MASLD or on previously published associations with NAFLD [32]. Model 1 was adjusted for age (y), sex, examination year and energy intake (kcal/d). Model 2 was adjusted for model 1 and leisure-time physical activity (kcal/d), smoking (never smoker, previous smoker, current smoker <20 cigarettes/d, and current smoker ≥20 cigarettes/d), intake of alcohol (g/wk), CVD, years of education, income (euros), and use of drugs with potential effects on liver fat accumulation. Model 3 was adjusted for model 2 and serum PUFAs (proportion of all serum fatty acids), and intakes of SFAs [percent of energy intake (E%)], protein (E%), fiber (g/d) and vitamin E (mg/d). Serum concentration instead of dietary intake for PUFAs was used because serum PUFAs have strong inverse associations with hepatic steatosis in this study population [32], and especially with the long-chain n-3 PUFAs the serum concentrations better reflect dietary intakes than the intakes estimated with 4-d food records [33]. In additional analyses, instead of the adjustment for the individual dietary factors in model 3, we adjusted model 2 further for the Baltic Sea Diet Score, as an indicator for the overall diet quality. All quantitative variables were entered as continuous variables in the models. Missing values in covariates were replaced with means of the study population (*n* = 2 in cigarette pack-years, *n* = 1 in years of education, *n* = 10 in serum PUFAs, and *n* = 99 in income). Statistical significance of the potential interactions by sex, BMI (<30 or ≥30), and glucose metabolism disturbances (presence of type 2 diabetes or metabolic syndrome, or impaired fasting glucose, yes or no) was assessed by stratified analysis and likelihood ratio tests using a multiplicative interaction term. All *P* values were 2-tailed (*α* = 0.05).

## Results

### Baseline characteristics

The mean ± SD age of the cohort was 63.1 ± 6.4 y at baseline in 1998–2001. Females composed 57% of the cohort. Data on the participants’ lifestyle factors, diseases, and dietary intakes in the whole population and according to the total dietary fructose intake are given in Table 1 . Those with higher fructose intake were more likely to be females, had a higher education and were less likely to smoke or have diabetes. Intakes of protein, saturated fatty acids, PUFAs and MUFAs, red meat, and dairy were lower, and the overall diet quality (Baltic Sea Diet Score) and intakes of carbohydrate, fiber, and fruit were higher in those with higher fructose intake.

**TABLE 1:** Baseline characteristics according to total dietary fructose intake^1^

Characteristic	The whole population	Fructose intake quartile (g/d)			
		1 (<25)	2 (25–32)	3 (33–39)	4 (>39)
Number of subjects	1531	382	383	383	383
Age (y)	63.1 ± 6.4	63.3 ± 6.3	63.5 ± 6.5	63.6 ± 6.4	62.0 ± 6.4
Sex, female (%)	56.5	38.2	60.3	67.1	60.3
Education (y)	9.5 ± 3.4	8.9 ± 3.0	9.2 ± 3.5	9.9 ± 3.6	10.1 ± 3.5
Income (€/y)	15,693 ± 10,528	15,567 ± 9619	15,178 ± 10,114	15,859 ± 11,899	16,168 ± 10,355
Leisure-time physical activity (kcal/d)	189.2 ± 205.1	178.0 ± 192.1	193.9 ± 207.6	194.3 ± 234.2	190.6 ± 183.2
Baltic Sea Diet Score^2^	12.6 ± 3.9	11.4 ± 3.7	12.3 ± 4.0	13.1 ± 3.7	13.7 ± 3.9
Current smoker (%)	10.0	15.7	9.9	7.6	6.8
Diabetes (%)	11.1	17.0	12.0	8.9	6.5
Metabolic syndrome (%)	29.6	32.5	32.7	26.4	26.6
Cardiovascular disease (%)	44.0	42.9	47.8	45.4	39.9
Hypertension (%)	64.5	64.9	68.4	64.8	59.8
Alcohol intake (g/wk)	24.7 ± 33.4	33.5 ± 38.5	23.1 ± 30.9	19.8 ± 29.0	22.6 ± 32.8
Medication use (%)^3^	41.0	39.8	40.7	41.0	42.6
Serum n-6 PUFAs (%)	31.9 ± 3.6	32.1 ± 3.7	31.9 ± 3.5	32.0 ± 3.5	31.7 ± 3.9
Serum n-3 PUFAs (%)	6.0 ± 1.8	6.0 ± 1.7	6.1 ± 1.8	6.1 ± 1.7	6.0 ± 1.8
Components of the FLI					
BMI (kg/m^2^)	27.8 ± 4.5	28.1 ± 4.5	28.1 ± 4.4	27.6 ± 4.7	27.2 ± 2.3
Waist circumference (cm)	91.7 ± 12.1	94.8 ± 11.8	92.0 ± 11.8	89.9 ± 12.6	89.9 ± 11.6
Triglycerides (mmol/L)	1.3 ± 0.7	1.3 ± 0.7	1.3 ± 0.7	1.3 ± 0.7	1.3 ± 0.7
GGT (U/L)	25.4 ± 25.3	27.9 ± 24.5	24.3 ± 20.1	24.9 ± 23.1	24.5 ± 32.0
Dietary intakes					
Energy (kcal/d)	1814 ± 556	1934 ± 603	1696 ± 514	1685 ± 499	1940 ± 550
Protein (E%)	17.3 ± 2.9	18.5 ± 3.0	17.9 ± 2.8	17.2 ± 2.7	15.7 ± 2.5
SFAs (E%)	14.1 ± 3.3	15.3 ± 3.5	14.3 ± 3.2	13.7 ± 2.9	12.9 ± 3.0
PUFAs (E%)	4.9 ± 1.4	5.3 ± 1.4	5.0 ± 1.5	4.8 ± 1.4	4.5 ± 1.2
MUFAs (E%)	10.9 ± 2.4	12.1 ± 2.4	11.0 ± 2.3	10.5 ± 2.1	9.8 ± 2.2
Trans fatty acids (E%)	0.9 ± 0.4	1.0 ± 0.4	0.9 ± 0.4	1.0 ± 0.4	0.9 ± 0.3
Carbohydrates (E%)	48.0 ± 6.3	43.5 ± 6.0	46.8 ± 5.3	49.3 ± 4.7	52.4 ± 5.3
Fiber (g/d)	22.5 ± 6.4	21.9 ± 7.0	22.7 ± 6.1	22.6 ± 5.4	22.9 ± 7.1
Vitamin E (mg/d)	8.3 ± 2.1	8.3 ± 2.2	8.3 ± 2.0	8.4 ± 2.0	8.3 ± 2.3
Red meat and game (g/d)	102 ± 72	132 ± 89	101 ± 66	83 ± 51	91 ± 67
Dairy (g/d)	485 ± 266	568 ± 292	475 ± 249	458 ± 251	439 ± 251
Coffee (g/d)	391 ± 228	427 ± 245	398 ± 214	363 ± 198	378 ± 246
Sources of fructose					
Added sugar (g/d)	20 ± 16	14 ± 12	19 ± 13	20 ± 14	28 ± 20
Added fructose (g/d)	0.04 ± 0.5	0.003 ± 0.06	0.04 ± 0.4	0 ± 0	0.1 ± 0.9
Syrup (g/d)	0.6 ± 1.7	0.4 ± 1.1	0.6 ± 1.3	0.7 ± 1.7	0.8 ± 2.4
Honey (g/d)	2.0 ± 7.2	0.5 ± 2.4	1.0 ± 3.7	2.2 ± 5.6	4.3 ± 12.2
Fruits (g/d)	105 ± 106	59 ± 63	86 ± 79	118 ± 96	157 ± 142
Berries (g/d)	46 ± 56	25 ± 35	44 ± 53	50 ± 55	63 ± 68
Juice (g/d)	134 ± 184	56 ± 114	88 ± 112	131 ± 133	260 ± 262
Soft drinks (g/d)	34 ± 93	23 ± 59	27 ± 77	32 ± 84	54 ± 132
Jams (g/d)	13 ± 34	5 ± 10	9 ± 13	12 ± 15	27 ± 61
Sweets and candies (g/d)	3 ± 11	2 ± 6	2 ± 6	3 ± 10	6 ± 17

Abbreviations: E%, percent of energy intake; FLI, fatty liver index; GGT, gamma-glutamyl transferase.

1 Values are means ± SD or percentages.

2 Baltic Sea Diet Score is an indicator of overall diet quality [26]. The score includes 9 components: Nordic fruits and berries, vegetables, cereals, low-fat and fat-free milk, fish, processed meat, ratio of PUFAs to saturated and trans fatty acids, total fat, and alcohol. The score ranges from 0 to 25, with higher score indicating better adherence to a healthy Nordic diet.

3 Drugs with potential effects on liver fat accumulation. These include drugs that were used by ≥1 participant: aspirin, ibuprofen, acetaminophen, corticosteroids, losartan, naproxen, methotrexate, omeprazole, pentoxifylline, valproic acid, amiodarone, tamoxifen, and tetracycline.

The mean total ± SD fructose intake was 33.0 ± 13.4 g/d, which was 7.4% (± 2.8%) of the total daily energy intake. Of the total fructose intake, 30.9 % (10.2 g/d) came from added or naturally present sucrose. The major dietary sources of fructose were sweeteners (mostly added sugar), fruits and berries, and beverages (Table 2). Supplemental Table 1 shows the population characteristics in quartiles of fructose from sweeteners. Those with higher intake of fructose from sweeteners were older, less educated, had less diabetes and metabolic syndrome and their BMI was lower. The fructose intakes based on sex, BMI, and presence or absence of glucose metabolism disturbances are shown in the Supplemental Tables 2–4. Intake of total fructose and fructose from fruits and berries and from sweets and candies was higher in females than in males (Supplemental Table 2), and the intake of fructose from sweeteners and added sugar was lower and fructose from beverages was higher among those with the BMI ≥30 than among those with the BMI <30 (Supplemental Table 3). There were no statistically significant differences in fructose intake based on the presence or absence of glucose metabolism disturbances (Supplemental Table 4).

**TABLE 2:** Intake of fructose from different dietary sources

Source	g/d	% of total intake
Total fructose intake	33.0 ± 13.4^1^	
Sweeteners	11.4 ± 3.7	34.5
Added sugar	10.2 ± 6.7	31.0
Added fructose	0.04 ± 0.5	0.1
Syrup	0.3 ± 0.7	2.1
Honey	0.9 ± 3.0	2.6
Fruits and berries	6.7 ± 5.5	20.2
Fruits	5.2 ± 5.3	15.7
Berries	1.5 ± 1.8	4.5
Beverages	6.3 ± 7.8	18.9
Juice	5.2 ± 7.3	15.7
Soda	1.1 ± 2.8	3.2
Jams	2.7 ± 6.7	8.3
Sweets and candies	0.6 ± 2.1	1.9
Other^2^	5.3 ± 2.6	16.1

1 Values are mean ± SD.

2 Includes fructose from, for example, vegetables, roots, and grains.

### Fructose intake, FLI, and MASLD in cross-sectional analyses

Table 3 shows the unadjusted mean values of FLI and multivariable-adjusted odds for MASLD in quartiles of fructose intake in cross-sectional analyses. Higher total fructose intake was associated with lower mean FLI. After adjustment for age, sex, examination year, and energy intake (model 1), the odds ratio for MASLD was 33% lower [95% confidence interval (CI): 8%, 52%] in the highest compared with the lowest quartile (*P*-trend across the quartiles = 0.01). Furthermore, adjustment for potential confounders (model 2) had little impact on the associations, but adjustment for other dietary factors strengthened the association (model 3). Among the sources of fructose, the strongest inverse associations were observed with fructose coming from sweeteners (Table 3). The odds ratio for MASLD was 60% lower (95% CI: 37%, 75%) in the highest compared with lowest quartile (model 3, *P*-trend < 0.001). Fructose from fruits and berries or from beverages was not associated with odds for MASLD (Table 3). The associations were in general similar when fructose intake was assessed as the percentage of energy intake (Supplemental Table 5), or when the Baltic Sea Diet Score was adjusted for instead of the individual dietary factors in model 3 (Supplemental Table 6).

**TABLE 3:** Mean FLI and odds for metabolic MASLD in quartiles of fructose intake in 1998–2001

	Fructose intake quartile				*P* for trend
	1 (*n* = 382)	2 (*n* = 383)	3 (*n* = 383)	4 (*n* = 383)	
Total fructose (g/d)	<24.6	24.6–32.1	32.2–39.7	>39.7	
Unadjusted mean FLI	47.1 (1.4)^1^	42.9 (1.4)	39.6 (1.4)	37.6 (1.4)	<0.001
Odds for MASLD (*n* of cases)	131	112	102	90	
Model 1	1	0.80 (0.59–1.10)^2^	0.73 (0.53–1.01)	0.67 (0.48–0.92)	0.01
Model 2	1	0.83 (0.60–1.14)	0.76 (0.55–1.06)	0.70 (0.50–0.97)	0.03
Model 3	1	0.80 (0.55–1.18)	0.74 (0.50–1.11)	0.58 (0.37–0.91)	0.02
Fructose from sweeteners^3^ (g/d)	<6.8	6.8–10.4	10.4–15.0	>15.0	
Unadjusted mean FLI	49.0 (1.4)	41.8 (1.4)	38.3 (1.4)	38.0 (1.3)	<0.001
Odds for MASLD (*n* of cases)	146	108	94	87	
Model 1	1	0.62 (0.45–0.84)	0.52 (0.37–0.71)	0.47 (0.34–0.65)	<0.001
Model 2	1	0.64 (0.47–0.88)	0.53 (0.38–0.73)	0.48 (0.34–0.67)	<0.001
Model 3	1	0.58 (0.40–0.85)	0.44 (0.30–0.65)	0.40 (0.26–0.61)	<0.001
Fructose from added sugar (g/d)	<6.1	6.1–9.4	9.4–13.2	>13.2	
Unadjusted mean FLI	47.7 (1.4)	43.0 (1.4)	38.2 (1.4)	38.3 (1.3)	<0.001
Odds for MASLD (*n* of cases)	138	116	91	90	
Model 1	1	0.75 (0.55–1.03)	0.55 (0.40–0.76)	0.54 (0.39–0.74)	<0.001
Model 2	1	0.76 (0.55–1.04)	0.57 (0.41–0.79)	0.52 (0.37–0.72)	<0.001
Model 3	1	0.78 (0.54–1.14)	0.50 (0.33–0.74)	0.42 (0.27–0.64)	<0.001
Fructose from fruits and berries (g/d)	<2.6	2.6–5.6	5.6–9.3	>9.3	
Unadjusted mean FLI	46.7 (1.4)	40.8 (1.4)	39.8 (1.4)	39.8 (1.5)	0.002
Odds for MASLD (*n* of cases)	131	102	98	104	
Model 1	1	0.74 (0.54–1.01)	0.72 (0.52–1.00)	0.83 (0.60–1.15)	0.38
Model 2	1	0.77 (0.56–1.06)	0.76 (0.55–1.06)	0.91 (0.65–1.27)	0.75
Model 3	1	0.73 (0.50–1.07)	0.71 (0.48–1.04)	0.92 (0.61–1.40)	0.82
Fructose from beverages^4^ (g/d)	<1.2	1.2–4.1	4.1–9.2	>9.2	
Unadjusted mean FLI	41.9 (1.4)	40.6 (1.4)	42.4 (1.4)	42.3 (1.4)	0.61
Odds for MASLD (*n* of cases)	101	112	107	115	
Model 1	1	1.15 (0.81–1.63)	1.06 (0.76–1.48)	1.24 (0.90–1.72)	0.26
Model 2	1	1.18 (0.83–1.69)	1.04 (0.74–1.47)	1.22 (0.88–1.70)	0.37
Model 3	1	1.30 (0.86–1.97)	1.15 (0.77–1.72)	1.35 (0.91–2.03)	0.25
Fructose from juices (g/d)	<0.7	0.7–2.7	2.7–7.7	>7.7	
Unadjusted mean FLI	44.1 (1.3)	40.7 (1.5)	40.7 (1.4)	41.6 (1.4)	0.43
Odds for MASLD (*n* of cases)	110	113	101	111	
Model 1	1	1.02 (0.72–1.44)	0.90 (0.64–1.27)	1.05 (0.76–1.45)	0.81
Model 2	1	1.05 (0.74–1.50)	0.89 (0.63–1.26)	1.02 (0.73–1.42)	0.98
Model 3	1	1.10 (0.73–1.67)	1.00 (0.66–1.50)	1.20 (0.81–1.79)	0.42

Model 1 adjusted for age, sex, examination year, and energy intake.

Model 2 adjusted for model 1 and leisure-time physical activity (kcal/d), smoking (never smoker, previous smoker, current smoker <20 cigarettes/d, and current smoker ≥ cigarettes/d), intake of alcohol (g/wk), history of cardiovascular diseases, years of education, income (euros), and drugs with potential effects on liver fat accumulation.

Model 3 adjusted for model 2 and serum PUFAs (proportion of all serum fatty acids), and intakes of SFAs E%, protein (E%), fiber (g/d), and vitamin E (mg/d).

Abbreviations: CI, confidence interval; E%, percent of energy intake; FLI, fatty liver index; MASLD, metabolic dysfunction**–**associated steatotic liver disease.

1 Values are means ± SEM from the analysis of variance.

2 Values are odds ratios (95% CI) from logistic regression.

3 Group “sweeteners” includes added sugar (89% of the total intake of sweeteners), fructose, syrup, and honey.

4 Group “beverages” includes juices and sodas.

In the stratified analyses based on sex, total fructose intake was inversely associated with the odds for MASLD only among the females but not in males, and fructose from beverages was associated with higher odds only among the males (Supplemental Figure 1). Although the *P* value for interaction indicated a difference in the associations between the males and females for fructose intake from fruits and berries (*P* = 0.04), the associations with odds for MASLD were not statistically significant. In the stratified analyses based on BMI, total fructose and fructose from fruits and berries were associated with the lower odds for MASLD among those with BMI ≥30 and fructose from sweeteners and added sugar was associated with lower odds among those with BMI <30, although not all interactions were statistically significant (Supplemental Figure 2). Fructose from beverages was associated with higher odds for MASLD only among those with BMI<30 (*P*-interaction = 0.03) (Supplemental Figure 2). Presence or absence of glucose metabolism disturbances did not modify the associations (*P*-interactions > 0.37, other data not shown).

### Fructose intake, FLI and MASLD in longitudinal analyses

Table 4 shows the mean values of FLI and odds for MASLD in 2005–2008 in quartiles of fructose intake assessed in 1998–2001. In these analyses, total fructose intake was not associated with MASLD. As in the cross-sectional analyses (Table 3), fructose from sweeteners had a strong inverse association with odds for MASLD: those in the highest compared with lowest quartile had 78% lower (95% CI: 49%, 90%) odds ratio for MASLD (model 3, *P*-trend < 0.001). Also similar to the cross-sectional analyses, fructose from fruits and berries or from beverages was not associated with odds for MASLD. The associations were again in general similar when fructose intake was assessed as the percentage of energy intake (Supplemental Table 7), or when model 3 included the Baltic Sea Diet Score instead of the individual dietary factors (Supplemental Table 8).

**TABLE 4:** Mean FLI and odds for MASLD in 2005–2008 in quartiles of fructose intake assessed in 1998–2001

	Fructose intake quartile				*P* for trend
	1 (*n* = 191)	2 (*n* = 192)	3 (*n* = 192)	4 (*n* = 192)	
Total fructose (g/d)	<26.3	26.3–34.2	34.2–41.2	>41.2	
Unadjusted mean FLI	36.7 (1.6)^1^	34.0 (1.6)	29.3 (1.4)	31.2 (1.6)	0.005
Odds for MASLD (*n* of cases)	30	32	15	22	
Model 1	1	1.14 (0.66–2.00)^2^	0.49 (0.25–0.96)	0.74 (0.41–1.36)	0.12
Model 2	1	1.16 (0.66–2.05)	0.48 (0.24–0.96)	0.77 (0.41–1.43)	0.15
Model 3	1	1.40 (0.77–2.55)	0.55 (0.27–1.13)	1.03 (0.50–2.16)	0.53
Fructose from sweeteners^3^ (g/d)	<7.5	7.6–11.0	11.0–15.5	>15.6	
Unadjusted mean FLI	38.3 (1.7)	32.9 (1.6)	30.7 (1.5)	29.1 (1.4)	<0.001
Odds for MASLD (*n* of cases)	40	24	20	15	
Model 1	1	0.57 (0.32–1.00)	0.46 (0.25–0.83)	0.32 (0.17–0.61)	<0.001
Model 2	1	0.57 (0.32–1.02)	0.44 (0.24–0.79)	0.31 (0.16–0.59)	<0.001
Model 3	1	0.52 (0.28–0.95)	0.35 (0.18–0.67)	0.24 (0.12–0.51)	<0.001
Fructose from added sugar (g/d)	<6.5	6.6–9.9	10.0–13.8	>13.8	
Unadjusted mean FLI	36.6 (1.7)	35.6 (1.5)	29.3 (1.5)	29.6 (1.4)	<0.001
Odds for MASLD (*n* of cases)	36	25	20	18	
Model 1	1	0.67 (0.38–1.18)	0.54 (0.29–0.97)	0.44 (0.24–0.81)	0.006
Model 2	1	0.67 (0.38–1.20)	0.51 (0.28–0.93)	0.41 (0.22–0.76)	0.003
Model 3	1	0.60 (0.33–1.10)	0.43 (0.23–0.83)	0.34 (0.17–0.69)	0.002
Fructose from fruits and berries (g/d)	<2.9	3.0–6.0	6.0–10.0	>10.0	
Unadjusted mean FLI	35.4 (1.6)	29.3 (1.5)	33.4 (1.5)	32.9 (1.6)	0.73
Odds for MASLD (*n* of cases)	31	13	26	29	
Model 1	1	0.40 (0.20–0.79)	0.86 (0.48–1.55)	1.01 (0.57–1.79)	0.39
Model 2	1	0.40 (0.20–0.82)	0.93 (0.51–1.71)	1.15 (0.62–2.13)	0.19
Model 3	1	0.39 (0.19–0.81)	0.99 (0.52–1.86)	1.42 (0.72–2.77)	0.06
Fructose from beverages^4^ (g/d)	<1.4	1.4–4.6	4.6–9.4	>9.4	
Unadjusted mean FLI	34.7 (1.6)	29.6 (1.5)	34.0 (1.6)	32.8 (1.6)	0.97
Odds for MASLD (*n* of cases)	28	18	27	26	
Model 1	1	0.64 (0.33–1.26)	0.98 (0.54–1.78)	0.93 (0.51–1.68)	0.76
Model 2	1	0.58 (0.29–1.16)	0.95 (0.51–1.74)	0.88 (0.48–1.62)	0.81
Model 3	1	0.58 (0.28–1.17)	1.04 (0.56–1.95)	1.00 (0.53–1.91)	0.51
Fructose from juices (g/d)	<0.8	0.8–3.3	3.3–7.8	>7.9	
Unadjusted mean FLI	35.3 (1.6)	29.9 (1.5)	33.3 (1.7)	32.6 (1.5)	0.71
Odds for MASLD (*n* of cases)	29	18	28	24	
Model 1	1	0.61 (0.31–1.22)	0.99 (0.55–1.80)	0.84 (0.46–1.54)	0.97
Model 2	1	0.60 (0.30–1.19)	0.95 (0.52–1.76)	0.85 (0.46–1.57)	0.91
Model 3	1	0.61 (0.30–1.24)	0.97 (0.52–1.82)	0.99 (0.52–1.88)	0.56

Model 1 adjusted for age, sex, examination year, and energy intake.

Model 2 adjusted for model 1 and leisure-time physical activity (kcal/d), smoking (never smoker, previous smoker, current smoker <20 cigarettes/d, and current smoker ≥ cigarettes/d), intake of alcohol (g/wk), history of cardiovascular diseases, years of education, income (euros), and drugs with potential effects on liver fat accumulation.

Model 3 adjusted for model 2 and serum PUFAs (proportion of all serum fatty acids), and intakes of SFAs E%, protein (E%), fiber (g/d), and vitamin E (mg/d).

Abbreviations: CI, confidence interval; E%, percent of energy intake; FLI, fatty liver index; MASLD, metabolic dysfunction**–**associated steatotic liver disease.

1 Values are means ± SEM from the analysis of variance.

2 Values are odds ratios (95% CI) from logistic regression.

3 Group “sweeteners” includes added sugar (89% of the total intake of sweeteners), fructose, syrup, and honey.

4 Group “beverages” includes juices and sodas.

In the stratified analyses based on sex, fructose from sweeteners and from added sugar had a stronger inverse association with odds for MASLD among the females than among the males, although the *P* value for interaction was not statistically significant for fructose from total sucrose (Supplemental Figure 3). No statistically significant interactions were observed in the analyses stratified by the BMI, although, as in the cross-sectional analyses, the associations of fructose from sweeteners and added sugar with lower odds for MASLD were statistically significant only among those with BMI <30 and the association of fructose from beverages with higher odds was statistically significant only among those with higher BMI (Supplemental Figure 4). Similar to the cross-sectional analyses, the presence or absence of glucose metabolism disturbances did not modify the associations (*P*-interactions >0.34, other data not shown).

## Discussion

In this population-based study among middle-aged and older males and females from Eastern Finland, higher fructose intake was associated with lower odds for prevalence of MASLD, supporting our hypothesis. However, in contrast to our hypothesis, especially fructose from sweeteners had a strong inverse association with odds for MASLD, in both cross-sectional and longitudinal analyses. Fructose from other sources was not associated with odds for MASLD.

Some experimental research has suggested that excess fructose intake may be linked with liver fat accumulation. In a controlled feeding trial, fructose restriction among overweight subjects with FLI > 60 led to a small but statistically significant decrease in intrahepatic lipid content [15]. In other studies, consuming fructose-sweetened beverages increased hepatic lipid content and visceral adiposity, promoted dyslipidemia, and decreased insulin sensitivity [10,12]. Furthermore, excess consumption of fructose-containing sugars was found to increase intrahepatocellular lipids in hypercaloric trials [13,14], but isocaloric exchange of fructose for other carbohydrates did not affect the intrahepatic lipids [14]. Similarly, in 1 controlled feeding trial of 10 healthy nonobese male volunteers with no MASLD, intake of 150 g of fructose over 4 h increased hepatic fat content only when coadministered with a high-fat load [34]. These results indicate that the excess energy intake rather than fructose specifically would be the reason for the increase in hepatic fat content. However, when 150 g of glucose was administered with the high-fat load, no increase in hepatic fat content was reported [34]. This would suggest that, when combined with high energy load, fructose may have more detrimental effects on hepatic fat content compared with glucose.

The amounts of fructose in controlled feeding trials have been substantially higher than what is commonly consumed, which makes it difficult to generalize the findings from these studies to free-living populations. For example, the median increase in fructose intake in hypercaloric trials was 193 g/d (IQR 158–211 g/d) and in isocaloric trials 182 g/d (IQR 115–204 g/d) [14]. In observational studies, the mean fructose intakes have ranged between 22 g/d and 48 g/d [[16], [17], [18], [19]]. Perhaps because of this reason, the epidemiological evidence is to some extent at odds with the evidence from the feeding trials. Observational studies from the Netherlands, Germany or Iran have found no association between total fructose intake and higher intrahepatic lipid content, fatty liver, or NAFLD [[16], [17], [18]], whereas our study and another Finnish study [19] found an inverse association. The mean energy-adjusted fructose intake in the other Finnish study was lower (about 22 g/d) compared with our study (about 33 g/d) and to the other studies (ranging from 32 g/d to 48 g/d). However, there is no information on the sources of fructose in that study, so we cannot confirm whether specific sources of fructose could explain the different findings between the Finnish studies and the studies from other countries. Altogether, our results together with other observational data suggest that fructose in moderate amounts would not appear to increase risk of MASLD, but instead the harms may only become apparent at very high intakes, as observed in experimental studies.

When different sources of fructose were investigated, we found the strongest inverse association between fructose intake and risk for MASLD with fructose coming from sweeteners, which were mainly added sugar. In our cohort, those with higher fructose intake from sweeteners had lower prevalence of type 2 diabetes and metabolic syndrome and lower BMI and waist circumference, compared with those with lower intake. This may explain the inverse association with lower odds of MASLD, as type 2 diabetes, metabolic syndrome and abdominal obesity are major risk factors for hepatic fat accumulation. Because waist circumference and BMI are components in the FLI calculation equation, they could not be adjusted for in the analyses to investigate that to what extent they might explain the association. However, in other studies, high intake of added sugar has been associated with increased adiposity, weight gain, increase in waist circumference, and obesity [35,36]. The average intake of added sugar in our cohort was significantly lower (mean intake 20 g/d) than in the previous studies (ranging from 78 to 100 g/d [35,36]) which may explain the different association with obesity.

In our study, fructose from beverages was not associated with risk of MASLD. This contrasts with the findings of the previous studies where especially fructose coming from beverages, such as sugar-sweetened or high-fructose corn syrup-sweetened beverages and juice, has been associated with increased hepatic lipid content [16,17]. The discrepant findings are possibly explained by the low use of soft drinks and high-fructose corn syrup in our cohort, though it is possible that high-fructose corn syrup was not used at all at that time. Also, the juice group includes berry juices, which are commonly homemade and consumed in Finland and have a different nutrient profile compared with fruit juices.

In the stratified analysis, sex and BMI modified some associations, but these results should be interpreted with caution as there were a large number of analyses, increasing the possibility of chance findings. If multiple comparisons were accounted for, only the cross-sectional association between total fructose intake and lower odds for MASLD in females but not in males would be considered a statistically significant interaction. However, there are no apparent explanations for this difference, as, that is, there were no major differences in fructose intake between the males and females.

It is possible that some lifestyle or nutritional factors that are associated with fructose intake could explain the association between fructose from sweeteners and lower risk of MASLD. One explanation could be lower saturated fat intake with increasing fructose intake, as high intake of SFAs is associated with an increase in liver fat [37]. However, in our study adjusting the analyses for saturated fat intake did not affect the associations and, on the other hand, there was no significant difference in saturated fat intake in quartiles of fructose intake from sweeteners. There were no differences in physical activity between the fructose intake categories, either, or in most other lifestyles or nutritional factors that could explain the findings. Alcohol intake was lower in the high fructose intake groups, but alcohol was also adjusted for in the analyses.

It is possible that Finland, especially in these older age groups, has different sweetener use habits than many other countries, which could explain the inverse association between fructose from sweeteners and MASLD. In our cohort, consumption of fructose from sweeteners did not seem to be consistently associated with poorer diet quality (Supplemental Table 1). For example, there was no significant association with the Baltic Sea Diet Score that describes adherence to a healthy Nordic diet. In contrast, higher consumption of fructose from sweeteners was associated with higher consumption of berries, and limited evidence suggests that intake of berries and the bioactive compounds in berries could have beneficial effects on liver health [38,39].

The strengths of this study include the availability of information on fructose intake from several different dietary sources, ability to control for several important confounders, inclusion of both males and females, and data from several time points, which enabled also longitudinal analyses. The weakness of this study is that we had to use the FLI algorithm to assess MASLD, rather than using imaging methods or liver biopsy, which are considered the reference standard for diagnosing hepatic steatosis [40]. However, the diagnostic performance of FLI for assessing hepatic steatosis has been described as good [41]. Dietary intake was assessed with a 4-d food record, which may not fully represent the typical dietary habits of individuals [42]. In addition, our study population included only middle-aged and older Caucasian males and females, so the findings may not be generalizable to younger populations or other ethnicities. Finally, although several potential lifestyle and dietary factors were accounted for in the statistical analyses, possibility of residual confounding cannot be excluded. Reverse causation is another potential concern especially in the cross-sectional analyses, but less likely to affect the longitudinal analyses.

In conclusion, in our study, fructose intake was not associated with MASLD and especially fructose from sweeteners was associated with lower odds for prevalent and incident MASLD. According to the recent guidelines for MASLD from European authorities [43], following a healthy diet (such as a Mediterranean diet), avoiding high intake of sugary drinks, ultraprocessed foods (rich in sugar and saturated fat) and red meat, and weight loss or avoidance of weight gain are important dietary strategies in the prevention and management of MASLD. Our study suggests that a moderate amount of fructose in the diet, even from added sugar, does not appear to increase risk of MASLD. However, on the basis of experimental studies, very high fructose intake should be avoided.

## Author contributions

The authors’ responsibilities were as follows – RHS: drafted the manuscript; HEKV, SH, JTS, T-PT, JKV: designed research; HEKV, SH, JTS, T-PT, JKV: conducted research; RHS, JKV: had full access to all the data in the study; JKV: takes responsibility for the integrity of the data and the accuracy of the data analysis; and all authors: provided critical revision of the manuscript for important intellectual content and approved the final manuscript.

## Data availability

The data will not be openly available because it contains sensitive personal information of the participants that cannot be completely anonymized.

## Funding

The KIHD study was for the most part funded by research grants to JTS. The funders had no role in the design, implementation, analysis, or interpretation of the data. The current study did not receive any specific grant from funding agencies or the public, commercial, or not-for-profit sectors.

## Conflict of interest

The authors report no conflicts of interest.

## References

[1] Rinella M.E., Lazarus J.V., Ratziu V., Francque S.M., Sanyal A.J., Kanwal F. (2024). A multisociety Delphi consensus statement on new fatty liver disease nomenclature, Ann. Hepatol..

[2] Hagström H., Vessby J., Ekstedt M., Shang Y. (2024). 99% of patients with NAFLD meet MASLD criteria and natural history is therefore identical. J. Hepatol..

[3] Younossi Z.M., Koenig A.B., Abdelatif D., Fazel Y., Henry L., Wymer M. (2016). Global epidemiology of nonalcoholic fatty liver disease-meta-analytic assessment of prevalence, incidence, and outcomes. Hepatology..

[4] Miao L., Targher G., Byrne C.D., Cao Y.Y., Zheng M.H. (2024). Current status and future trends of the global burden of MASLD. Trends Endocrinol. Metab..

[5] Younossi Z.M., Golabi P., Paik J.M., Henry A., Van Dongen C., Henry L. (2023). The global epidemiology of nonalcoholic fatty liver disease (NAFLD) and nonalcoholic steatohepatitis (NASH): a systematic review. Hepatology.

[6] Younossi Z.M., Henry L. (2021). Epidemiology of non-alcoholic fatty liver disease and hepatocellular carcinoma. JHEP Rep.

[7] Xiao J., Ng C.H., Chan K.E., Fu C., Tay P., Yong J.N. (2023). Hepatic, extra-hepatic outcomes and causes of mortality in NAFLD - an umbrella overview of systematic review of meta-analysis. J. Clin. Exp. Hepatol..

[8] George E.S., Forsyth A., Itsiopoulos C., Nicoll A.J., Ryan M., Sood S. (2018). Practical dietary recommendations for the prevention and management of nonalcoholic fatty liver disease in adults. Adv Nutr.

[9] Herman M.A., Birnbaum M.J. (2021). Molecular aspects of fructose metabolism and metabolic disease. Cell Metab..

[10] Stanhope K.L., Schwarz J.M., Keim N.L., Griffen S.C., Bremer A.A., Graham J.L. (2009). Consuming fructose-sweetened, not glucose-sweetened, beverages increases visceral adiposity and lipids and decreases insulin sensitivity in overweight/obese humans. J. Clin. Investig..

[11] Hieronimus B., Medici V., Bremer A.A., Lee V., Nunez M.V., Sigala D.M. (2020). Synergistic effects of fructose and glucose on lipoprotein risk factors for cardiovascular disease in young adults. Metabolism.

[12] Sigala D.M., Hieronimus B., Medici V., Lee V., Nunez M.V., Bremer A.A. (2021). Consuming sucrose- or HFCS-sweetened beverages increases hepatic lipid and decreases insulin sensitivity in adults. J. Clin. Endocrinol. Metab..

[13] Lee D., Chiavaroli L., Ayoub-Charette S., Khan T.A., Zurbau A., Au-Yeung F. (2022). Important food sources of fructose-containing sugars and non-alcoholic fatty liver disease: a systematic review and meta-analysis of controlled trials. Nutrients.

[14] Chiu S., Sievenpiper J.L., de Souza R.J., Cozma A.I., Mirrahimi A., Carleton A.J. (2014). Effect of fructose on markers of non-alcoholic fatty liver disease (NAFLD): a systematic review and meta-analysis of controlled feeding trials. Eur. J. Clin. Nutr..

[15] Simons N., Veeraiah P., Simons P.I.H.G., Schaper N.C., Kooi M.E., Schrauwen-Hinderling V.B. (2021). Effects of fructose restriction on liver steatosis (FRUITLESS); a double-blind randomized controlled trial. Am. J. Clin. Nutr..

[16] Buziau A.M., Eussen S.J.P.M., Kooi M.E., van der Kallen C.J.H., van Dongen M.C.J.M., Schaper N.C. (2022). Fructose intake from fruit juice and sugar-sweetened beverages is associated with higher intrahepatic lipid content: the Maastricht Study. Diabetes Care.

[17] Weber K.S., Simon M.C., Strassburger K., Markgraf D.F., Buyken A.E., Szendroedi J. (2018). Habitual fructose intake relates to insulin sensitivity and fatty liver index in recent-onset type 2 diabetes patients and individuals without diabetes. Nutrients.

[18] Afsharfar M., Salimi Z., Aminnezhad Kavkani B., Shekari S., Abbastorki S., Majidi N. (2023). Association of nonalcoholic fatty liver disease with the different types of dietary carbohydrates: a cross-sectional study. J. Diabetes Metab. Disord..

[19] Kanerva N., Sandboge S., Kaartinen N.E., Männistö S., Eriksson J.G. (2014). Higher fructose intake is inversely associated with risk of nonalcoholic fatty liver disease in older Finnish adults. Am. J. Clin. Nutr..

[20] DiStefano J.K., Shaibi G.Q. (2021). The relationship between excessive dietary fructose consumption and paediatric fatty liver disease. Pediatr. Obes..

[21] OʼSullivan T.A., Oddy W.H., Bremner A.P., Sherriff J.L., Ayonrinde O.T., Olynyk J.K. (2014). Lower fructose intake may help protect against development of nonalcoholic fatty liver in adolescents with obesity. J. Pediatr. Gastroenterol. Nutr..

[22] Liu W., Zhai D., Zhang T., Mudoti N.G., Chang Q., Liu Y. (2023). Meta-analysis of the association between major foods with added fructose and non-alcoholic fatty liver disease. Food Funct.

[23] Salonen J.T. (1988). Is there a continuing need for longitudinal epidemiologic research? The Kuopio Ischaemic Heart Disease Risk Factor Study. Ann. Clin. Res..

[24] Virtanen J.K., Mursu J., Tuomainen T.P., Voutilainen S. (2014). Dietary fatty acids and risk of coronary heart disease in men: the Kuopio Ischemic Heart Disease Risk Factor Study, Arterioscler. Thromb. Vasc. Biol..

[25] Willett W., Willett W. (2013). Nutritional epidemiology. Monographs in epidemiology and biostatistics.

[26] Tertsunen H.M., Hantunen S., Tuomainen T.P., Virtanen J.K. (2020). Healthy Nordic diet and risk of disease death among men: the Kuopio Ischaemic Heart Disease Risk Factor Study. Eur. J. Nutr..

[27] Salonen J.T., Nyyssönen K., Korpela H., Tuomilehto J., Seppänen R., Salonen R. (1992). High stored iron levels are associated with excess risk of myocardial infarction in eastern Finnish men. Circulation.

[28] Laaksonen D.E., Lakka T.A., Lakka H.M., Nyyssönen K., Rissanen T., Niskanen L.K. (2002). Serum fatty acid composition predicts development of impaired fasting glycaemia and diabetes in middle-aged men. Diabet. Med..

[29] López-Pascual E., Rienda I., Perez-Rojas J., Rapisarda A., Garcia-Llorens G., Jover R. (2024). Drug-Induced Fatty Liver Disease (DIFLD): a comprehensive analysis of clinical, biochemical, and histopathological data for mechanisms identification and consistency with current adverse outcome pathways. Int. J. Mol. Sci..

[30] Lakka T.A., Venäläinen J.M., Rauramaa R., Salonen R., Tuomilehto J., Salonen J.T. (1994). Relation of leisure-time physical activity and cardiorespiratory fitness to the risk of acute myocardial infarction. N. Engl. J. Med..

[31] Bedogni G., Bellentani S., Miglioli L., Masutti F., Passalacqua M., Castiglione A. (2006). The Fatty Liver Index: a simple and accurate predictor of hepatic steatosis in the general population. BMC Gastroenterol.

[32] Mäkelä T.N.G., Tuomainen T.P., Hantunen S., Virtanen J.K. (2022). Associations of serum n-3 and n-6 polyunsaturated fatty acids with prevalence and incidence of nonalcoholic fatty liver disease. Am. J. Clin. Nutr..

[33] Virtanen J.K., Mursu J., Voutilainen S., Uusitupa M., Tuomainen T.P. (2014). Serum omega-3 polyunsaturated fatty acids and risk of incident type 2 diabetes in men: the Kuopio Ischemic Heart Disease Risk Factor study. Diabetes Care.

[34] Dusilová T., Kovář J., Drobný M., Šedivý P., Dezortová M., Poledne R. (2019). Different acute effects of fructose and glucose administration on hepatic fat content. Am. J. Clin. Nutr..

[35] Chiavaroli L., Cheung A., Ayoub-Charette S., Ahmed A., Lee D., Au-Yeung F. (2023). Important food sources of fructose-containing sugars and adiposity: a systematic review and meta-analysis of controlled feeding trials. Am. J. Clin. Nutr..

[36] Endy E.J., Yi S.Y., Steffen B.T., Shikany J.M., Jacobs D.R., Goins R.K. (2024). Added sugar intake is associated with weight gain and risk of developing obesity over 30 years: the CARDIA study. Nutr. Metab. Cardiovasc. Dis..

[37] Berná G., Romero-Gomez M. (2020). The role of nutrition in non-alcoholic fatty liver disease: pathophysiology and management. Liver Int.

[38] Książek E., Goluch Z., Bochniak M. (2024). Vaccinium spp. Berries in the prevention and treatment of non-alcoholic fatty liver disease: a comprehensive update of preclinical and clinical research. Nutrients.

[39] Li H.Y., Gan R.Y., Shang A., Mao Q.Q., Sun Q.C., Wu D.T. (2021). Plant-based foods and their bioactive compounds on fatty liver disease: effects, mechanisms, and clinical application. Oxid. Med. Cell Longev..

[40] Castera L., Friedrich-Rust M., Loomba R. (2019). Noninvasive assessment of liver disease in patients with nonalcoholic fatty liver disease. Gastroenterology.

[41] Fedchuk L., Nascimbeni F., Pais R., Charlotte F., Housset C., Ratziu V. (2014). Performance and limitations of steatosis biomarkers in patients with nonalcoholic fatty liver disease, Aliment. Pharmacol. Ther..

[42] Willet W., Sampson L., Willet W. (2013). Nutritional epidemiology.

[43] European Association for the Study of the Liver (EASL) (2024). European Association for the Study of Diabetes (EASD), European Association for the Study of Obesity (EASO), EASL-EASD-EASO clinical practice guidelines on the management of Metabolic Dysfunction-Associated Steatotic Liver Disease (MASLD). J. Hepatol..

