# Supplementary material for: Associations of Fructose Consumption with Prevalence and Incidence of Metabolic Dysfunction–Associated Steatotic Liver Disease—The Kuopio Ischaemic Heart Disease Risk Factor Study
Source: J Nutr. 2026 Jan 6;156(2):101318. doi: 10.1016/j.tjnut.2025.101318 (PMC12975370; doi:10.1016/j.tjnut.2025.101318)
Supplement: multimedia component 1 [file mmc1.pdf]

ONLINE SUPPLEMENTARY MATERIAL

Associations of fructose consumption with prevalence and incidence of metabolic dysfunction-associated steatotic liver disease (MASLD) - the Kuopio Ischaemic Heart Disease Risk Factor Study

Saarinen RH, et al.

CONTENTS

Page 2. Supplemental Table 1. Baseline characteristics according to dietary intake of fructose from sweeteners.

Page 4. Supplemental Table 2. Intake of fructose from different dietary sources stratified by sex.

Page 5. Supplemental Table 3. Intake of fructose from different dietary sources stratified by the body mass index.

Page 6. Supplemental Table 4. Intake of fructose from different dietary sources stratified by the presence or absence of glucose metabolism disturbances.

Page 7. Supplemental Table 5. Mean fatty liver index and odds for metabolic dysfunction-associated steatotic liver disease in quartiles of fructose intake as percentage of total energy intake in 1998-2001.

Page 9. Supplemental Table 6. Odds for metabolic dysfunction-associated steatotic liver disease in quartiles of fructose intake in 1998-2001, adjusted for overall diet quality (Baltic Sea Diet Score).

Page 10. Supplemental Table 7. Mean fatty liver index and odds for metabolic dysfunction-associated steatotic liver disease in 2005-2008 in quartiles of fructose intake as percentage of total energy intake assessed in 1998-2001.

Page 12. Supplemental Table 8. Odds for metabolic dysfunction-associated steatotic liver disease in 2005-2008 in quartiles of fructose intake assessed in 1998-2001, adjusted for overall diet quality (Baltic Sea Diet Score)

Page 13. Supplemental Figure 1. Odds for metabolic dysfunction-associated steatotic liver disease for each 5 g increase in fructose intake in 1998-2001, stratified by sex.

Page 14. Supplemental Figure 2. Odds for metabolic dysfunction-associated steatotic liver disease for each 5 g increase in fructose intake in 1998-2001, stratified by the body mass index.

Page 15. Supplemental Figure 3. Odds for metabolic dysfunction-associated steatotic liver disease in 2005-2008 for each 5 g increase in fructose intake assessed in 1998-2001, stratified by sex.

Page 16. Supplemental Figure 4. Odds metabolic dysfunction-associated steatotic liver disease in 2005-2008 for each 5 g increase in fructose intake assessed in 1998-2001, stratified by the body mass index.

SUPPLEMENTAL TABLE 1. Baseline characteristics according to dietary intake of fructose from sweeteners[1]

Characteristic	The whole population	Intake quartile of fructose from sweeteners (g/day)			
		1 (<7)	2 (7-10)	3 (10-15)	4 (>15)
Number of subjects	1531	382	383	383	383
Age (years)	63.1 ± 6.4	61.6 ± 6.1	62.8 ± 6.5	63.7 ± 6.6	64.2 ± 6.2
Sex, female (%)	56.5	44.0	65.8	68.7	47.5
Education (y)	9.5 ± 3.4	9.9 ± 3.4	9.8 ± 3.7	9.5 ± 3.4	8.9 ± 3.2
Income (€/y)	15693 ± 10528	17392 ± 11271	16030 ± 10328	15170 ± 11531	14185 ± 8487
Leisure-time physical activity (kcal/d)	189.2 ± 205.1	187.0 ± 198.0	200.1 ± 198.4	190.0 ± 226.4	179.7 ± 196.4
Baltic Sea Diet Score[2]	12.6 ± 3.9	12.8 ± 3.9	12.6 ± 3.9	12.8 ± 3.8	12.4 ± 4.0
Current smoker (%)	10.0	12.6	8.9	6.5	12.0
Diabetes (%)	11.1	19.9	12.8	7.3	4.4
Metabolic syndrome (%)	29.6	36.7	29.8	29.7	22.1
Cardiovascular disease (%)	44.0	43.5	44.9	44.6	43.1
Hypertension (%)	64.5	69.6	63.7	64.5	60.1
Alcohol intake (g/wk)	24.7 ± 33.4	33.7 ± 37.6	22.6 ± 30.6	20.6 ± 31.4	22.1 ± 31.9
Medication use[3]	41.9	42.7	41.3	42.6	37.6
Serum n-6 polyunsaturated fatty acids (%)	31.9 ± 3.6	31.9 ± 3.7	31.9 ± 3.6	31.9 ± 3.7	32.0 ± 3.7
Serum n-3 polyunsaturated fatty acids (%)	6.0 ± 1.8	6.1 ± 1.8	6.2 ± 1.7	6.1 ± 1.8	5.8 ± 1.7
Components of the fatty liver index					
Body mass index, kg/m^2	27.8 ± 4.5	28.5 ± 4.5	28.2 ± 4.6	27.5 ± 4.4	26.9 ± 4.2
Waist circumference, cm	91.7 ± 12.1	95.1 ± 11.8	91.4 ± 12.2	89.4 ± 11.9	90.7 ± 11.8
Triglycerides, mmol/L	1.3 ± 0.7	1.3 ± 0.7	1.3 ± 0.8	1.3 ± 0.7	1.3 ± 0.6
Gamma-glutamyl transferase, U/L	25.4 ± 25.3	30.2 ± 38.0	24.7 ± 19.1	23.2 ± 21.6	23.4 ± 16.2
Dietary intakes					
Energy (kcal/d)	1814 ± 556	1954 ± 557	1651 ± 511	1667 ± 520	1984 ± 549
Protein (E%)[4]	17.3 ± 2.9	18.3 ± 3.1	18.1 ± 2.8	17.2 ± 2.6	15.7 ± 2.4
Saturated fatty acids (E%)	14.1 ± 3.3	14.3 ± 3.6	13.8 ± 3.2	13.9 ± 3.0	14.2 ± 3.3
Polyunsaturated fatty acids (E%)	4.9 ± 1.4	5.2 ± 1.4	4.9 ± 1.3	4.8 ± 1.4	4.9 ± 1.4
Monounsaturated fatty acids (E%)	10.9 ± 2.4	11.4 ± 2.6	10.8 ± 2.4	10.5 ± 2.4	10.7 ± 2.2
Trans fatty acids (E%)	0.9 ± 0.4	0.9 ± 0.4	0.9 ± 0.4	1.0 ± 0.4	1.0 ± 0.4
Carbohydrates (E%)	48.0 ± 6.3	45.5 ± 6.6	47.5 ± 6.2	49.0 ± 5.7	50.0 ± 5.7

Fiber (g/d)	22.5 ± 6.4	23.9 ± 7.9	22.3 ± 5.9	22.5 ± 5.4	21.4 ± 6.1
Vitamin E (mg/d)	8.3 ± 2.1	8.5 ± 2.2	8.2 ± 1.8	8.4 ± 2.0	8.2 ± 2.4
Red meat and game (g/d)	102 ± 72	127 ± 83	99 ± 68	86 ± 67	96 ± 60
Dairy (g/d)	485 ± 266	516 ± 283	468 ± 238	457 ± 250	499 ± 286
Coffee (g/d)	391 ± 228	419 ± 246	383 ± 209	344 ± 190	419 ± 253
Sources of fructose					
Added sugar (g/d)	20 ± 16	8 ± 7	14 ± 8	21 ± 9	38 ± 17
Added fructose (g/d)	0.04 ± 0.5	0.01 ± 0.1	0.01 ± 0.2	0.05 ± 0.4	0.08 ± 0.9
Syrup (g/d)	0.6 ± 1.7	0.5 ± 1.1	0.5 ± 1.1	0.6 ± 1.3	1.0 ± 2.7
Honey (g/d)	2.0 ± 7.2	0.4 ± 1.9	0.5 ± 1.9	1.5 ± 4.0	5.6 ± 12.9
Fruits (g/d)	105 ± 106	121 ± 123	109 ± 106	103 ± 96	86 ± 96
Berries (g/d)	46 ± 56	37 ± 54	39 ± 56	50 ± 53	57 ± 58
Juice (g/d)	134 ± 184	151 ± 230	128 ± 167	120 ± 152	137 ± 177
Soft drinks (g/d)	34 ± 93	42 ± 100	35 ± 104	25 ± 66	34 ± 96
Jams (g/d)	13 ± 34	14 ± 57	12 ± 20	13 ± 23	13 ± 20
Sweets and candies (g/d)	3 ± 11	4 ± 16	3 ± 8	3 ± 9	3 ± 9

[1]Values are means ± SD or percentages.
[2]Baltic Sea Diet Score is an indicator of overall diet quality. The score includes nine components: Nordic fruits and berries, vegetables, cereals, low-fat and fat-free milk, fish, processed meat, ratio of polyunsaturated fatty acids to saturated and trans fatty acids, total fat, and alcohol. The score ranges from 0 to 25, with higher score indicating better adherence to a healthy Nordic diet.
[3]Drugs with potential effects on liver fat accumulation. These include drugs that were used by at least one participant: aspirin, ibuprofen, acetaminophen, corticosteroids, losartan, naproxen, methotrexate, omeprazole, pentoxifylline, valproic acid, amiodarone, tamoxifen and tetracycline.
[4]E%, percent of energy.

SUPPLEMENTAL TABLE 2. Intake of fructose from different dietary sources, stratified by sex

Source	Female	Male
Total fructose intake	34.6 ± 10.7	30.8 ± 16.0*
Sweeteners (added sugar, added fructose, syrup, and honey)	11.4 ± 6.0	11.3 ± 8.8
Added sugar	10.1 ± 5.3	10.4 ± 8.2
Added fructose	0.04 ± 0.6	0.03 ± 0.4
Syrup	0.3 ± 0.6	0.2 ± 0.8
Honey	1.0 ± 2.7	0.7 ± 3.4
Fruits and berries	7.5 ± 5.3	5.6 ± 5.6*
Fruits	5.8 ± 5.2	4.4 ± 5.3*
Berries	1.7 ± 1.9	1.2 ± 1.7*
Beverages (juices and sodas)	6.4 ± 6.5	6.0 ± 9.2
Juice	5.4 ± 6.0	4.9 ± 8.8
Soda	1.0 ± 2.6	1.1 ± 3.1
Jams	3.0 ± 3.7	2.4 ± 9.2
Sweets and candies	0.8 ± 2.5	0.4 ± 1.6*
Other sources[1]	5.5 ± 2.4	5.1 ± 2.8*

*P for difference <0.05.

[1]Includes fructose from, for example, vegetables, roots, and grains.

SUPPLEMENTAL TABLE 3. Intake of fructose from different dietary sources, stratified by the body mass index

Source	BMI<30 (kg/m²)	BMI ≥30 (kg/m²)
Total fructose intake	33.3 ± 13.6	32.2 ± 12.7
Sweeteners (added sugar, added fructose, syrup, and honey)	11.8 ± 7.5	10.3 ± 6.8*
Added sugar	10.6 ± 6.9	9.2 ± 5.9*
Added fructose	0.0 ± 0.3	0.1 ± 0.8
Syrup	0.3 ± 0.7	0.3 ± 0.6
Honey	0.9 ± 3.0	0.7 ± 3.1
Fruits and berries	6.7 ± 5.4	6.7 ± 5.8
Fruits	5.2 ± 5.2	5.2 ± 5.6
Berries	1.5 ± 1.8	1.4 ± 2.0
Beverages (juices and sodas)	6.0 ± 7.5	6.9 ± 8.6*
Juice	5.1 ± 7.1	5.6 ± 7.9
Soda	1.0 ± 2.3	1.3 ± 3.9
Jams	2.9 ± 7.5	2.4 ± 3.4
Sweets and candies	0.6 ± 1.6	0.8 ± 3.3
Other sources[1]	5.3 ± 2.6	5.2 ± 2.4

*P for difference <0.05.

[1]Includes fructose from, for example, vegetables, roots, and grains.

BMI, body mass index.

SUPPLEMENTAL TABLE 4. Intake of fructose from different dietary sources, stratified by the presence or absence of glucose metabolism disturbances[1]

Source	No	Yes
Total fructose intake	33.9 ± 13.3	32.7 ± 13.4
Sweeteners (added sugar, added fructose, syrup, and honey)	11.7 ± 8.0	11.3 ± 7.1
Added sugar	10.4 ± 7.2	10.2 ± 6.5
Added fructose	0.0 ± 0.3	0.0 ± 0.6
Syrup	0.2 ± 0.5	0.3 ± 0.7
Honey	1.1 ± 3.7	0.8 ± 2.8
Fruits and berries	6.8 ± 5.8	6.6 ± 5.4
Fruits	5.4 ± 5.6	5.1 ± 5.2
Berries	1.4 ± 1.7	1.5 ± 1.8
Beverages (juices and sodas)	6.1 ± 7.9	6.3 ± 7.7
Juice	5.2 ± 7.5	5.2 ± 7.3
Soda	0.9 ± 2.8	1.1 ± 2.9
Jams	2.8 ± 4.6	2.7 ± 7.3
Sweets and candies	0.8 ± 1.9	0.6 ± 2.2
Other sources[2]	5.7 ± 2.7	5.2 ± 2.5*

*P for difference <0.05.

[1]Defined as type 2 diabetes or metabolic syndrome, or impaired fasting glucose.

[2]Includes fructose from, for example, vegetables, roots, and grains.

SUPPLEMENTAL TABLE 5. Mean fatty liver index and odds for metabolic dysfunction-associated steatotic liver disease in quartiles of fructose intake as percentage of total energy intake in 1998-2001

	Fructose intake quartile				
	1 (n=382)	2 (n=383)	3 (n=383)	4 (n=383)	P for trend
**Total fructose (E%)**	<5.4	5.4-7.2	7.2-9.0	>9.1	
Unadjusted mean FLI	46.7 (1.4)[1]	42.9 (1.4)	40.3 (1.5)	37.3 (1.4)	<0.001
Odds for MASLD (n of cases)	132	110	104	89	
Model 1	1	0.80 (0.59-1.10)[2]	0.77 (0.56-1.06)	0.64 (0.46-0.88)	0.007
Model 2	1	0.82 (0.60-1.12)	0.81 (0.59-1.12)	0.66 (0.47-0.91)	0.02
Model 3	1	0.77 (0.53-1.12)	0.80 (0.54-1.19)	0.52 (0.33-0.83)	0.01
**Fructose from sweeteners[3] (E%)**	<1.3	1.3-2.2	2.2-3.3	>3.3	
Unadjusted mean FLI	48.0 (1.5)	43.0 (1.4)	38.4 (1.4)	37.7 (1.3)	<0.001
Odds for MASLD (n of cases)	148	109	93	85	
Model 1	1	0.65 (0.48-0.88)	0.54 (0.39-0.74)	0.45 (0.33-0.63)	<0.001
Model 2	1	0.66 (0.48-0.90)	0.55 (0.40-0.76)	0.45 (0.32-0.63)	<0.001
Model 3	1	0.63 (0.44-0.91)	0.46 (0.31-0.68)	0.37 (0.24-0.57)	<0.001
**Fructose from added sugar (E%)**	<1.2	1.2-2.0	2.0-3.0	>3.0	
Unadjusted mean FLI	46.8 (1.5)	43.5 (1.4)	38.5 (1.4)	38.3 (1.3)	<0.001
Odds for MASLD (n of cases)	140	115	91	89	
Model 1	1	0.76 (0.56-1.02)	0.57 (0.41-0.78)	0.52 (0.38-0.72)	<0.001
Model 2	1	0.78 (0.57-1.06)	0.58 (0.42-0.81)	0.51 (0.36-0.71)	<0.001
Model 3	1	0.77 (0.54-1.12)	0.53 (0.36-0.78)	0.41 (0.27-0.63)	<0.001
**Fructose from fruits and berries (E%)**	<0.6	0.6-1.3	1.3-2.2	>2.2	
Unadjusted mean FLI	46.2 (1.4)	41.0 (1.4)	41.3 (1.5)	38.6 (1.4)	<0.001
Odds for MASLD (n of cases)	131	96	112	96	
Model 1	1	0.68 (0.50-0.94)	0.88 (0.64-1.20)	0.69 (0.49-0.96)	0.10
Model 2	1	0.70 (0.50-0.96)	0.93 (0.67-1.29)	0.74 (0.52-1.05)	0.26
Model 3	1	0.66 (0.45-0.96)	0.85 (0.57-1.26)	0.65 (0.42-0.99)	0.12

**Fructose from beverages[4] (E%)**	<0.0	0.0-0.9	0.9-2.1	>2.1	
Unadjusted mean FLI	42.5 (1.3)	39.1 (1.6)	42.2 (1.4)	42.4 (1.4)	0.61
Odds for MASLD (n of cases)	142	68	109	116	
Model 1	1	0.80 (0.57-1.12)	0.98 (0.73-1.32)	1.09 (0.81-1.47)	0.30
Model 2	1	0.80 (0.57-1.13)	0.96 (0.71-1.30)	1.06 (0.79-1.44)	0.41
Model 3	1	0.84 (0.56-1.26)	1.03 (0.72-1.48)	1.14 (0.79-1.65)	0.31
**Fructose from juices (E%)**	<0.0	0.0-0.6	0.6-1.8	>1.8	
Unadjusted mean FLI	43.9 (1.1)	37.0 (2.1)	41.2 (1.4)	41.3 (1.4)	0.37
Odds for MASLD (n of cases)	184	40	97	114	
Model 1	1	0.72 (0.48-1.06)	0.81 (0.61-1.09)	1.00 (0.76-1.33)	0.73
Model 2	1	0.74 (0.50-1.12)	0.80 (0.60-1.08)	0.97 (0.73-1.30)	0.92
Model 3	1	0.79 (0.49-1.27)	0.82 (0.58-1.16)	1.11 (0.78-1.58)	0.43

[1]Values are means ± SEM from the analysis of variance.
[2]Values are odds ratios (95% confidence interval) from logistic regression.
[3]Group sweeteners includes added sugar (89% of the total intake of sweeteners), fructose, syrup, and honey.
[4]Group beverages includes juices and sodas.
FLI, fatty liver index; MASLD, metabolic dysfunction-associated steatotic liver disease.
Model 1 adjusted for age, sex, examination year and energy intake.
Model 2 adjusted for model 1 and leisure-time physical activity (kcal/d), smoking (never smoker, previous smoker, current smoker <20 cigarettes/d, and current smoker ≥ cigarettes/d), intake of alcohol (g/wk), history of cardiovascular diseases, years of education, income, and drugs with potential effects on liver fat accumulation.
Model 3 adjusted for model 2 and serum polyunsaturated fatty acids (proportion of all serum fatty acids), and intakes of saturated fatty acids (E%), protein (E%), fiber (g/d) and vitamin E (mg/d).

SUPPLEMENTAL TABLE 6. Odds for metabolic dysfunction-associated steatotic liver disease in quartiles of fructose intake in 1998-2001, adjusted for overall diet quality (Baltic Sea Diet Score)

	Fructose intake quartile				
	1 (n=382)	2 (n=383)	3 (n=383)	4 (n=383)	P for trend
**Total fructose (g/day)**	<24.6	24.6-32.1	32.2-39.7	>39.7	
n of cases	131	112	102	90	
Odds ratio (95% confidence interval)	1	0.85 (0.62-1.17)	0.80 (0.57-1.11)	0.74 (0.53-1.04)	0.08
**Fructose from sweeteners[1] (g/day)**	<6.8	6.8-10.4	10.4-15.0	>15.0	
n of cases	146	108	94	87	
Odds ratio (95% confidence interval)	1	0.63 (0.46-0.87)	0.52 (0.37-0.72)	0.46 (0.33-0.64)	<0.001
**Fructose from added sugar (g/day)**	<6.1	6.1-9.4	9.4-13.2	>13.2	
n of cases	138	116	91	90	
Odds ratio (95% confidence interval)	1	0.75 (0.54-1.03)	0.56 (0.40-0.77)	0.49 (0.35-0.68)	<0.001
**Fructose from fruits and berries (g/day)**	<2.6	2.6-5.6	5.6-9.3	>9.3	
n of cases	131	102	98	104	
Odds ratio (95% confidence interval)	1	0.82 (0.59-1.14)	0.87 (0.62-1.22)	1.10 (0.76-1.58)	0.43
**Fructose from beverages[2] (g/day)**	<1.2	1.2-4.1	4.1-9.2	>9.2	
n of cases	101	112	107	115	
Odds ratio (95% confidence interval)	1	1.18 (0.83-1.69)	1.05 (0.74-1.47)	1.21 (0.87-1.69)	0.39
**Fructose from juices (g/day)**	<0.7	0.7-2.7	2.7-7.7	>7.7	
n of cases	110	113	101	111	
Odds ratio (95% confidence interval)	1	1.03 (0.72-1.47)	0.88 (0.62-1.25)	1.02 (0.73-1.42)	0.99

[1]Group “sweeteners” includes added sugar (89% of the total intake of sweeteners), fructose, syrup, and honey.

[2]Group “beverages” includes juices and sodas.

Model 3 adjusted for age, sex, examination year, energy intake, leisure-time physical activity (kcal/d), smoking (never smoker, previous smoker, current smoker <20 cigarettes/d, and current smoker ≥ cigarettes/d), intake of alcohol (g/wk), history of cardiovascular diseases, years of education, income (euros), drugs with potential effects on liver fat accumulation, and the Baltic Sea Diet Score (excluding the alcohol component).

SUPPLEMENTAL TABLE 7. Mean fatty liver index and odds for metabolic dysfunction-associated steatotic liver disease in 2005-2008 in quartiles of fructose intake as percentage of total energy intake assessed in 1998-2001

	Fructose intake quartile				
	1 (n=191)	2 (n=192)	3 (n=192)	4 (n=192)	P for trend
**Total fructose (E%)**	<5.8	5.8-7.6	7.6-9.4	>9.4	
Unadjusted mean FLI	37.2 (1.6)[1]	33.8 (1.6)	28.7 (1.4)	31.4 (1.6)	0.002
Odds for MASLD (n of cases)	33	32	11	23	
Model 1	1	1.00 (0.59-1.72)[2]	0.31 (0.15-0.64)	0.69 (0.38-1.25)	0.05
Model 2	1	1.02 (0.59-1.76)	0.30 (0.14-0.63)	0.74 (0.40-1.36)	0.07
Model 3	1	1.16 (0.65-2.06)	0.35 (0.16-0.77)	0.92 (0.46-1.88)	0.31
**Fructose from sweeteners[3] (E%)**	<1.5	1.5-2.4	2.4-3.4	>3.4	
Unadjusted mean FLI	36.4 (1.7)	35.2 (1.6)	31.5 (1.5)	28.0 (1.4)	<0.001
Odds for MASLD (n of cases)	34	30	21	14	
Model 1	1	0.85 (0.49-1.45)	0.56 (0.31-1.01)	0.35 (0.18-0.68)	<0.001
Model 2	1	0.83 (0.48-1.44)	0.51 (0.28-0.93)	0.33 (0.16-0.66)	<0.001
Model 3	1	0.82 (0.46-1.44)	0.45 (0.23-0.85)	0.31 (0.15-0.64)	<0.001
**Fructose from added sugar (E%)**	<1.3	1.3-2.2	2.2-3.1	>3.1	
Unadjusted mean FLI	35.4 (1.7)	36.6 (1.6)	29.7 (1.5)	29.3 (1.5)	<0.001
Odds for MASLD (n of cases)	32	30	19	18	
Model 1	1	0.89 (0.51-1.54)	0.55 (0.30-1.02)	0.48 (0.25-0.90)	0.01
Model 2	1	0.89 (0.51-1.56)	0.50 (0.27-0.94)	0.44 (0.23-0.85)	0.005
Model 3	1	0.86 (0.48-1.53)	0.48 (0.25-0.92)	0.41 (0.20-0.84)	0.005
**Fructose from fruits and berries (E%)**	<0.6	0.6-1.4	1.4-2.3	>2.3	
Unadjusted mean FLI	35.9 (1.6)	31.2 (1.5)	30.7 (1.4)	33.3 (1.7)	0.44
Odds for MASLD (n of cases)	31	18	18	32	
Model 1	1	0.57 (0.30-1.07)	0.59 (0.31-1.12)	1.23 (0.67-2.23)	0.25
Model 2	1	0.59 (0.31-1.13)	0.63 (0.33-1.23)	1.40 (0.74-2.64)	0.13

Model 3	1	0.57 (0.29-1.10)	0.69 (0.34-1.39)	1.58 (0.79-3.18)	0.05
**Fructose from beverages[4] (E%)**	<0.0	0.0-1.0	1.0-2.1	>2.1	
Unadjusted mean FLI	33.2 (1.5)	31.3 (1.5)	33.5 (1.6)	32.8 (1.6)	0.90
Odds for MASLD (n of cases)	32	15	25	27	
Model 1	1	0.58 (0.30-1.11)	0.86 (0.49-1.52)	0.95 (0.54-1.65)	0.74
Model 2	1	0.56 (0.29-1.08)	0.82 (0.46-1.47)	0.95 (0.54-1.67)	0.72
Model 3	1	0.53 (0.27-1.04)	0.90 (0.49-1.64)	1.10 (0.59-2.02)	0.37
**Fructose from juices (E%)**	<0.0	0.0-0.7	0.7-1.8	>1.8	
Unadjusted mean FLI	33.5 (1.4)	29.9 (1.7)	34.4 (1.6)	31.9 (1.5)	0.71
Odds for MASLD (n of cases)	39	8	30	22	
Model 1	1	0.42 (0.19-0.93)	1.08 (0.64-1.82)	0.78 (0.44-1.36)	0.73
Model 2	1	0.40 (0.18-0.90)	1.08 (0.63-1.84)	0.78 (0.44-1.38)	0.77
Model 3	1	0.37 (0.16-0.85)	1.08 (0.62-1.87)	0.90 (0.49-1.64)	0.81

[1]Values are means ± SEM from the analysis of variance.
[2]Values are odds ratios (95% confidence interval) from logistic regression.
[3]Group sweeteners includes added sugar (89% of the total intake of sweeteners), fructose, syrup, and honey.
[4]Group beverages includes juices and sodas.
FLI, fatty liver index; MASLD, metabolic dysfunction-associated steatotic liver disease.
Model 1 adjusted for age, sex, examination year and energy intake.
Model 2 adjusted for model 1 and leisure-time physical activity (kcal/d), smoking (never smoker, previous smoker, current smoker <20 cigarettes/d, and current smoker ≥ cigarettes/d), intake of alcohol (g/wk), history of cardiovascular diseases, years of education, income, and drugs with potential effects on liver fat accumulation.
Model 3 adjusted for model 2 and serum polyunsaturated fatty acids (proportion of all serum fatty acids), and intakes of saturated fatty acids (E%), protein (E%), fiber (g/d) and vitamin E (mg/d).

SUPPLEMENTAL TABLE 8. Odds for metabolic dysfunction-associated steatotic liver disease in 2005-2008 in quartiles of fructose intake assessed in 1998-2001, adjusted for overall diet quality (Baltic Sea Diet Score)

	Fructose intake quartile				
	1 (n=191)	2 (n=192)	3 (n=192)	4 (n=192)	P for trend
**Total fructose (g/day)**	<26.3	26.3-34.2	34.2-41.2	>41.2	
n of cases	30	32	15	22	
Odds ratio (95% confidence interval)	1	1.17 (0.66-2.06)[2]	0.49 (0.25-0.98)	0.79 (0.42-1.48)	0.19
**Fructose from sweeteners[1] (g/day)**	<7.5	7.6-11.0	11.0-15.5	>15.6	
n of cases	40	24	20	15	
Odds ratio (95% confidence interval)	1	0.58 (0.32-1.03)	0.43 (0.24-0.79)	0.29 (0.15-0.57)	<0.001
**Fructose from added sugar (g/day)**	<6.5	6.6-9.9	10.0-13.8	>13.8	
n of cases	36	25	20	18	
Odds ratio (95% confidence interval)	1	0.68 (0.38-1.21)	0.50 (0.27-0.93)	0.39 (0.21-0.73)	0.002
**Fructose from fruits and berries (g/day)**	<2.9	3.0-6.0	6.0-10.0	>10.0	
n of cases	31	13	26	29	
Odds ratio (95% confidence interval)	1	0.44 (0.21-0.89)	1.08 (0.57-2.04)	1.42 (0.73-2.78)	0.07
**Fructose from beverages[2] (g/day)**	<1.4	1.4-4.6	4.6-9.4	>9.4	
n of cases	28	18	27	26	
Odds ratio (95% confidence interval)	1	0.57 (0.29-1.14)	0.95 (0.51-1.74)	0.85 (0.46-1.58)	0.88
**Fructose from juices (g/day)**	<0.8	0.8-3.3	3.3-7.8	>7.9	
n of cases	29	18	28	24	
Odds ratio (95% confidence interval)	1	0.59 (0.29-1.19)	0.94 (0.51-1.72)	0.83 (0.45-1.54)	0.97

[1]Group "sweeteners" includes added sugar (89% of the total intake of sweeteners), fructose, syrup, and honey.
[2]Group "beverages" includes juices and sodas.
Model 3 adjusted for age, sex, examination year, energy intake, leisure-time physical activity (kcal/d), smoking (never smoker, previous smoker, current smoker <20 cigarettes/d, and current smoker ≥ cigarettes/d), intake of alcohol (g/wk), history of cardiovascular diseases, years of education, income (euros), drugs with potential effects on liver fat accumulation, and the Baltic Sea Diet Score (excluding the alcohol component).

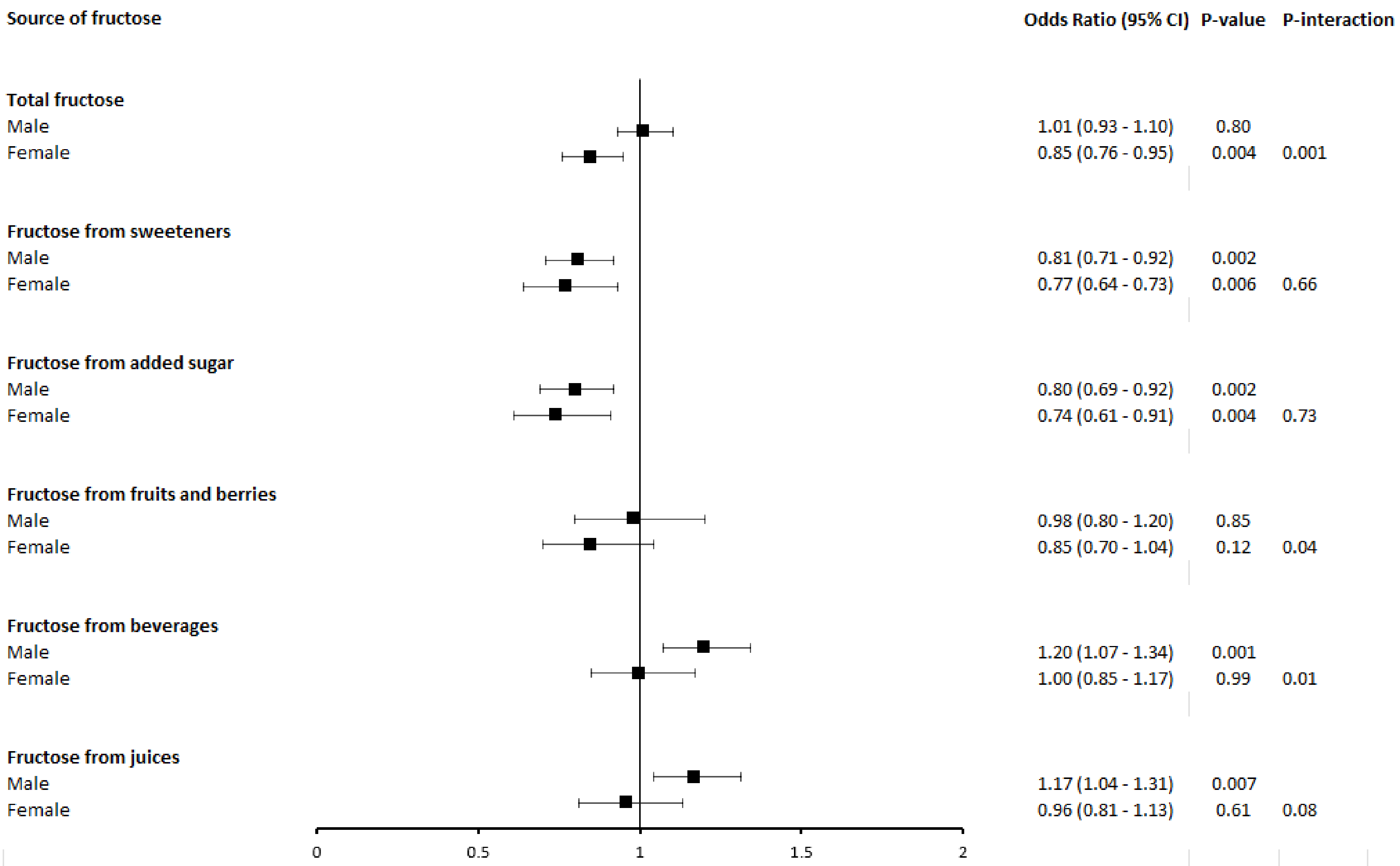

Supplemental Fig. 1. Odds for metabolic dysfunction-associated steatotic liver disease for each 5 g increase in fructose intake in 1998-2001, stratified by sex.

Source of fructose	Odds Ratio (95% CI)	P-value	P-interaction
**Total fructose**			
BMI <30	0.95 (0.85 - 1.05)	0.31	
BMI ≥30	0.85 (0.74 - 0.99)	0.04	0.25
**Fructose from sweeteners**			
BMI <30	0.70 (0.59 - 0.83)	0.001	
BMI ≥30	1.03 (0.80 - 1.33)	0.82	0.03
**Fructose from added sugar**			
BMI <30	0.73 (0.61 - 0.87)	0.001	
BMI ≥30	1.00 (0.75 - 1.33)	1.00	0.13
**Fructose from fruits and berries**			
BMI <30	1.01 (0.82 - 1.26)	0.92	
BMI ≥30	0.74 (0.56 - 0.98)	0.04	0.05
**Fructose from beverages**			
BMI <30	1.17 (1.02 - 1.34)	0.03	
BMI ≥30	0.93 (0.77 - 1.13)	0.46	0.03
**Fructose from juices**			
BMI <30	1.16 (1.01 - 1.33)	0.04	
BMI ≥30	0.88 (0.73 - 1.07)	0.19	0.02

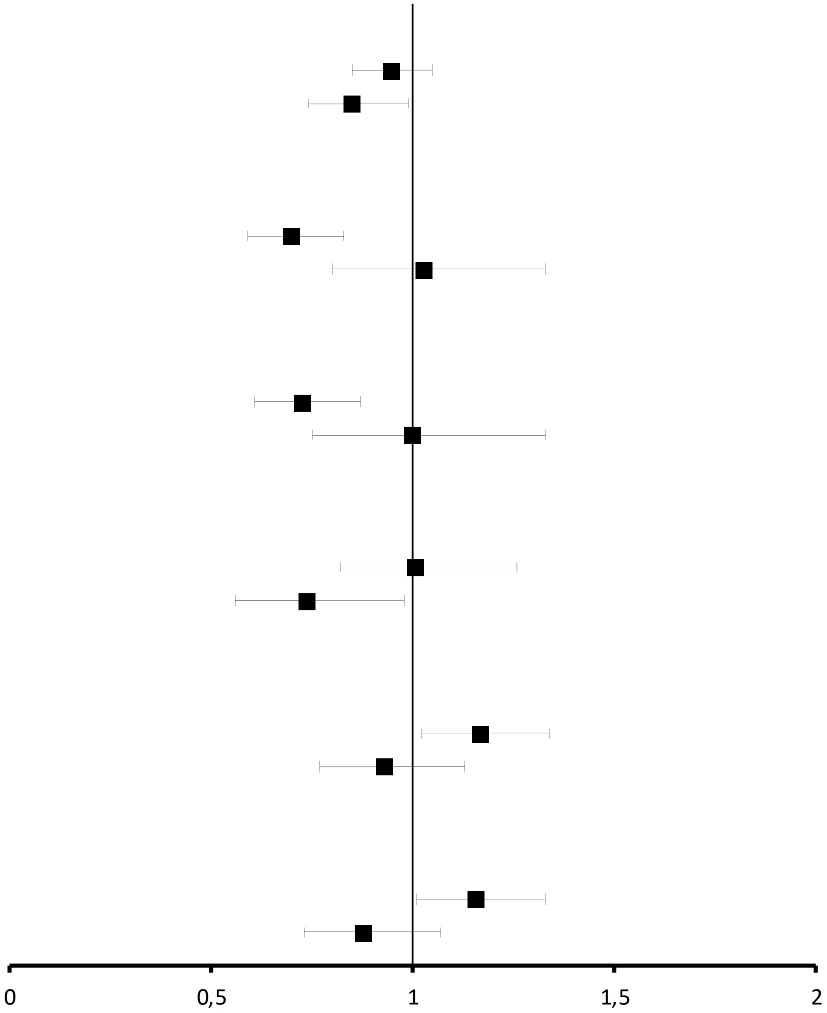

Supplemental Fig. 2. Odds for metabolic dysfunction-associated steatotic liver disease for each 5 g increase in fructose intake in 1998-2001, stratified by the body mass index (BMI).

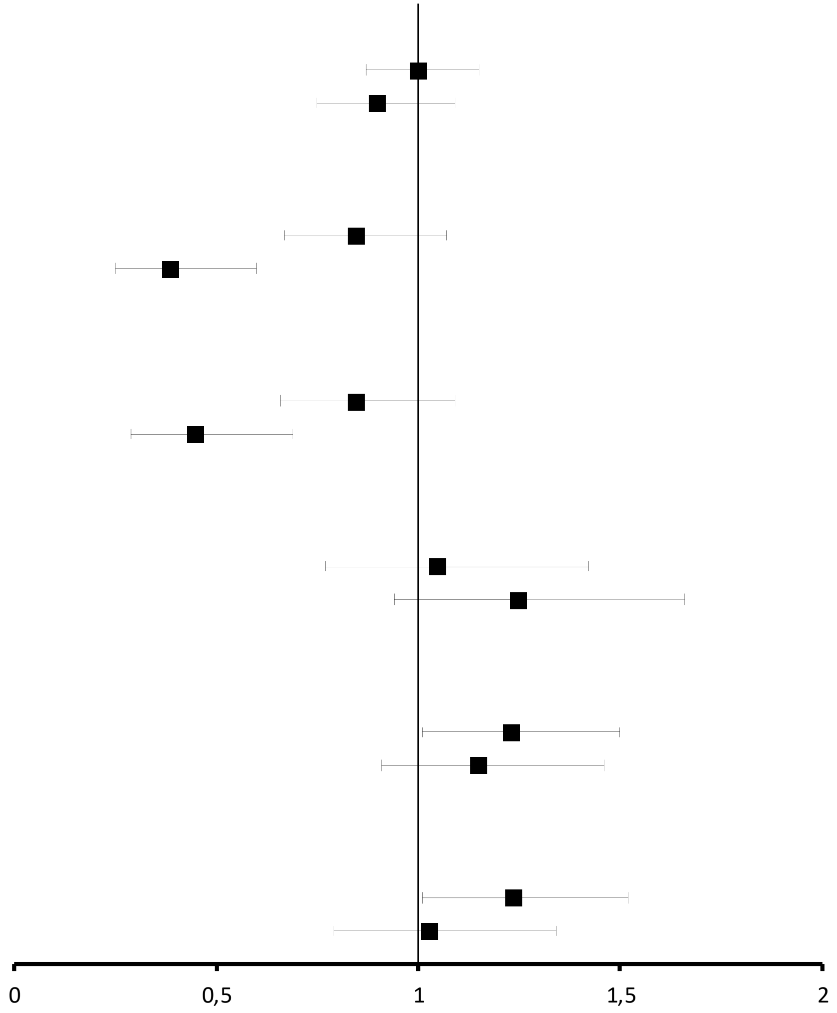

Source of fructose	Odds Ratio (95% CI)	P-value	P-interaction
**Total fructose**			
Male	1.00 (0.87 - 1.15)	0.99	
Female	0.90 (0.75 - 1.09)	0.30	0.56
**Fructose from sweeteners**			
Male	0.85 (0.67 - 1.07)	0.16	
Female	0.39 (0.25 - 0.60)	0.001	0.005
**Fructose from added sugar**			
Male	0.85 (0.66 - 1.09)	0.20	
Female	0.45 (0.29 - 0.69)	0.001	0.03
**Fructose from fruits and berries**			
Male	1.05 (0.77 - 1.42)	0.78	
Female	1.25 (0.94 - 1.66)	0.13	0.49
**Fructose from beverages**			
Male	1.23 (1.01 - 1.50)	0.24	
Female	1.15 (0.91 - 1.46)	0.04	0.81
**Fructose from juices**			
Male	1.24 (1.01 - 1.52)	0.04	
Female	1.03 (0.79 - 1.34)	0.83	0.39

Supplemental Fig. 3. Odds for metabolic dysfunction-associated steatotic liver disease in 2005-2008 for each 5 g increase in fructose intake assessed in 1998-2001, stratified by sex.

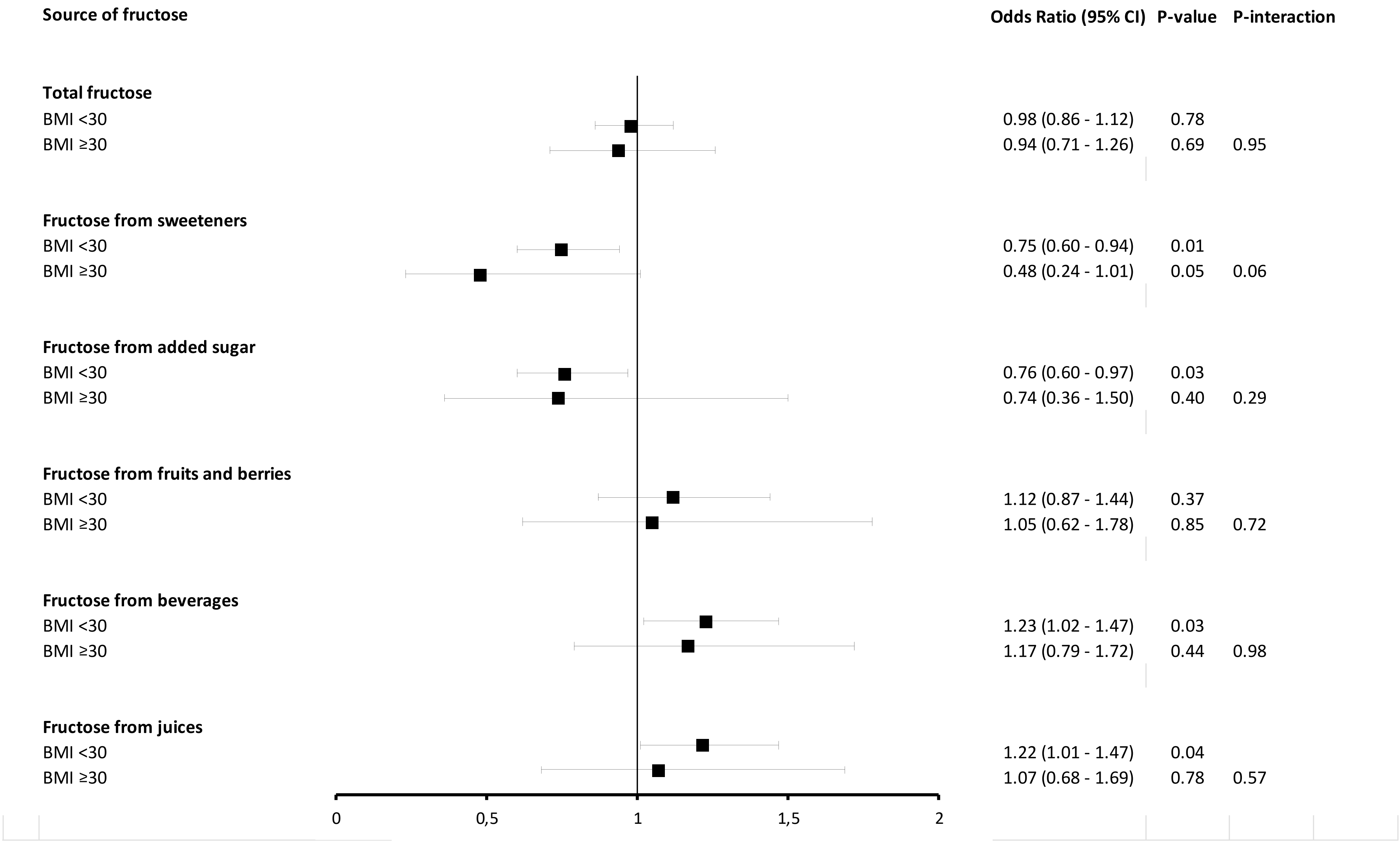

Supplemental Fig. 4. Odds for metabolic dysfunction-associated steatotic liver disease in 2005-2008 for each 5 g increase in fructose intake assessed in 1998-2001, stratified by the body mass index (BMI).
